# Biochemical and molecular evaluation of resveratrol and selenium nanoparticles in managing type 2 diabetes and its complications

**DOI:** 10.1038/s41598-025-11156-x

**Published:** 2025-07-15

**Authors:** Aya Y. Soliman, Nihal M. Elguindy, Abdulrahman M. Saleh, Mahmoud Balbaa

**Affiliations:** 1 Department of Biochemistry, Faculty of Science, Alexandria University, Alexandria, 21511 Egypt; 2 Department of Pharmaceutical Chemistry, Faculty of Pharmacy, Cairo University, Kasr El‑Aini Street, Cairo, 11562 Egypt; 3 Epidemiological Surveillance Unit, Aweash El-Hagar Family Medicine Center, MOHP, Mansoura, 35711 Egypt

**Keywords:** Insulin resistance, Diabetes, Inflammation, Apoptosis, Resveratrol, Selenium nanoparticle, Molecular Docking, Biochemistry, Molecular biology, Nanoscience and technology

## Abstract

The therapeutic potential of dietary polyphenols, such as resveratrol (Res) and elemental selenium nanoparticles (SeNPs), has gained increasing attention for the treatment and management of type 2 diabetes mellitus (T2DM). In this study, we investigated a novel therapeutic strategy using chitosan-stabilized Res/SeNPs (CS/Res/SeNPs) in a mouse model of T2DM induced by high-fat diet (HFD) feeding combined with multiple low-dose streptozotocin (STZ) injections. We evaluated the effects of these nanoparticles on glucose and insulin levels, oxidative/antioxidant balance, expression of apoptotic and anti-apoptotic genes, and inflammatory mediators using biochemical, histopathological, and molecular docking analyses. Treatment with free Res, CS/Res/SeNPs-5, CS/Res/SeNPs-10, and Metformin (Met) improved hyperglycemia, insulin resistance (IR), and dyslipidemia, and normalized elevated liver and kidney biomarkers. These treatments also exhibited anti-inflammatory, antioxidant, and anti-apoptotic effects. CS/Res/SeNPs-10 showed the most potent hepatoprotective and anti-diabetic effects. The docking results revealed that Res binds to key insulin signaling proteins, such as phosphatidylinositol 3-kinase (PI3K)/protein kinase B (AKT)/mammalian target of rapamycin (mTOR). Collectively, our findings highlight the therapeutic promise of CS/Res/SeNPs in managing T2DM complications and suggest a cost-effective strategy to enhance health outcomes and quality of life in diabetic patients.

**Supplementary Information:**

The online version contains supplementary material available at 10.1038/s41598-025-11156-x.

## Introduction

Type 2 diabetes mellitus (T2DM) is a complex metabolic condition characterized by IR and inadequate compensatory insulin production. Approximately 90–95% of diabetic cases are classified as T2DM^1^. The incidence of DM is expected to rise globally up to 643 million (11.3%) and 783 million (12.2%) by 2030 and 2045, respectively^2^. Low-grade inflammation and oxidative stress are key contributors to T2DM progression and its various complications^3^. Hyperlipidemia triggers the release of pro-inflammatory cytokines from adipose tissue, while hyperglycemia promotes oxidative stress via increasing reactive oxygen species (ROS) production and mitochondrial dysfunction. These effects collectively contribute to apoptosis, β-cell dysfunction, insulin deficiency, and the subsequent development of T2DM^4,5^. Pharmacological treatment is the mainstay of care for patients with T2DM, particularly with the advent of newer antidiabetic drug classes such as sodium-glucose cotransporter-2 (SGLT2) inhibitors and glucagon-like peptide-1 receptor (GLP-1R) agonists, which have shown benefits in mitigating cardiovascular and renal complications. These agents are often prescribed alongside metformin (Met) for at-risk individuals. However, long-term use of pharmacologic therapies may lead to undesirable side effects. Consequently, there is a growing interest in exploring natural products as safer alternatives, which may serve as adjunct dietary supplements to prevent or alleviate T2DM-related complications^6^.

Resveratrol (Res) is highly promising for numerous reasons. Its safety profile has been well-documented due to its long-standing use as a dietary supplement. It is a multi-target molecule that can influence several cellular signaling pathways. Res is readily available and economically viable due to its low price and extensive distribution^7^. Res is known for its anti-obesity, antidiabetic, anti-inflammatory, and antioxidant properties^8^. Owing to poor intestinal absorption, substantial metabolism, and excretion, Res has a limited oral bioavailability, which restricts its positive health effects^9^. To overcome the aforementioned challenges in administering Res and enhance its health-promoting bioactive properties, nanotechnology is being applied in the development of novel carriers for the effective delivery of Res. Res-loaded nanocarriers have demonstrated improved oral bioavailability and enhanced their bioactivities, including antioxidant, anti-inflammatory, anti-cancer, neuroprotective, cardioprotective, and wound-healing effects^10^.

Among various trace elements, selenium (Se) is an essential micronutrient metalloid that plays a crucial role in the prevention and treatment of various diseases^11^. Selenium is a vital component of several selenoproteins, including the antioxidant enzyme glutathione peroxidase (GPx). Epidemiological studies have linked Se deficiency with the onset of multiple diseases^11^. Recent studies suggested that selenium nanoparticles (SeNPs) possess unique antioxidant mechanisms, primarily through stimulating the expression and activity of selenoproteins, which protect cells and tissues from oxidative damage caused by free radicals^12^. Concerning toxicity, SeNPs exhibited seven-fold lower acute toxicity compared to selenite and three times less toxic than organic Se, when exposed to mice^13^. In addition, SeNPs exhibited potent anti-diabetic properties^14^. Through the potential antioxidant and anti-inflammatory features of Se and SeNPs, many studies have suggested a close association between a high serum level of Se and a lower risk of developing T2DM^14,15^.

It has been shown that the incorporation of Res into SeNPs enhances their biological activity. Various studies indicate that polysaccharides, when combined with Se, can improve biological processes and address relevant drawbacks. Natural polysaccharide-based nanoparticles can encapsulate therapeutic agents and prolong their in vivo retention time. Chitosan (CS), a naturally occurring polysaccharide, is widely utilized in nanomedicine and advanced drug delivery systems due to its biocompatibility, low toxicity, and capacity to enhance drug bioavailability and reduce pharmacological side effects. In the context of diabetes and metabolic disorders, CS has been shown to protect pancreatic β-cells, reduce hyperglycemia, and modulate lipid metabolism^16^. Furthermore, CS serves as an effective carrier for bioactive molecules such as Res and SeNPs, enhancing their stability, cellular uptake, and therapeutic efficacy. The positively charged amino groups of CS can react with negatively charged sodium tripolyphosphate (TPP) to encapsulate Res, forming stable CS/Res nanoparticles (CS/Res-NPs). Previous studies have demonstrated that CS-based Res/SeNP formulations improve neurocognitive function and exert antioxidant and anti-inflammatory effects in models of Alzheimer’s disease with metabolic comorbidities^17^. Our study applies this nanoformulation in the context of T2DM, where oxidative stress and inflammation are also central but less studied in relation to Res/SeNP formulations. Furthermore, our formulation incorporates CS not only as a structural carrier but as an active enhancer of drug delivery, capitalizing on its mucoadhesive characteristics, positive surface charge, and biodegradable nature.

Based on these principles, our study aims to assess a potent anti-diabetic drug with low toxicity using nanotechnology. This objective is addressed by investigating the molecular and biochemical effects of nanoparticle-based treatments using CS/Res/SeNPs at doses of 5 mg/kg and 10 mg/kg in a T2DM model. The outcomes are compared to those of the standard antidiabetic drug Met and conventional Res treatment. This study examines the mechanisms through which the synthesized nanoparticles CS/Res/Se-NPs affect oxidative stress, apoptosis, and inflammation in liver tissues to establish the optimal dosage and to substantiate the rationale for employing natural polyphenol/element nanoparticles in the prevention of diabetic complications. Additionally, we conducted **molecular docking** analyses to assess the binding of Res to key proteins involved in insulin signaling.

## Materials and methods

### Pharmacokinetic properties calculated using Swiss ADME

The pharmacokinetic properties were calculated using the **SwissADME** tool provided by the Swiss Bioinformatics Institute. This web-based platform enables the prediction of various ADME (Absorption, Distribution, Metabolism, and Excretion) parameters, along with potential target interactions. These predictions support the evaluation of resveratrol’s pharmaceutical properties and its suitability for clinical application.

### Network construction and analysis methods

The protein-protein interaction (PPI) data for Res targets were collected from the STRING database version 11.0 (https://string-db.org/). Functional pathways of Res were analyzed using gene ontology (GO) enrichment analysis based upon the database for Annotation, Visualization and Integrated Discovery (DAVID) version 6.8 (https://david.ncifcrf.gov/).

### Preparation of nanoparticles

CS powder (Sigma-Aldrich, USA) was dissolved in 1% acetic acid (30 mL) (Sigma-Aldrich, USA) to obtain 50 mg/mL CS solution, pH 4.6; afterward, 0.1 g of Res powder (Sigma-Aldrich, USA) was dissolved in 15 mL of ethanol (Sigma-Aldrich, USA). After that, the prepared Res solution was added to the CS solution. The mixture was then stirred for 20 min at 800 rpm to get the orange precipitate. Sodium selenite (0.172 g/20 mL) (Sigma-Aldrich, USA) and ascorbic acid (1.23 g/140 mL) (Sigma-Aldrich, USA) were prepared. Using a magnetic stirrer, the ascorbic acid solution started to be dropped wisely and added to the sodium selenite solution while being stirred until the solution turned red. After 20 min, TPP crosslinker (0.09 g/10 mL) (Sigma-Aldrich, USA) was added, and the solution was diluted to 500 mL and left to be stirred overnight. The room was maintained at a temperature of 22 ± 2.0℃.

### Characterization of nanoparticles

To fix the nanoparticles for transmission electron microscope (TEM) analysis, a drop of our synthesized nanoparticle suspension was applied to copper grids coated with carbon. A TEM (JEOL JEM-1400Flash instrument, Japan) with an operating voltage of 80 kV and a resolution of up to 2.4 Å was employed to record the pictures after the specimens had been dried using an infrared lamp. Additionally, the particle surface charge, often referred to as the zeta-potential, was assessed using Zetasizer (Malvern Instruments, UK) based on the dynamic light scattering (DLS) approach. The produced nanoparticles were further subjected to Fourier transform infrared (FTIR) spectroscopy (FTIR-8400 S Shimadzu, Japan).

### Animals

Fifty-four Swiss male albino mice aged 8 to 10 weeks, weighing 35 to 50 g obtained from the City of Scientific Research and Technological Applications, Alexandria University, Egypt. In a room with a 12-h day-night cycle, the mice were housed in polycarbonate cages (six groups), with nine mice per group. The room was maintained at a temperature of 22 ± 2.0℃, and a humidity of 45–46%. The mice were fed with a consistent commercial diet. Eight mice died throughout the entire experiment, and the remaining animals were sacrificed at the end of the experimental study. The deaths occurred randomly following STZ induction before the allocation of animals into treatment groups. All experimental procedures were carried out in accordance with the relevant guidelines and regulations according to the animal protocols approved by the Ethics Committee of the Faculty of Science, Alexandria University, Egypt (AU Approval No. 04231031102).

**Fig. 1:**
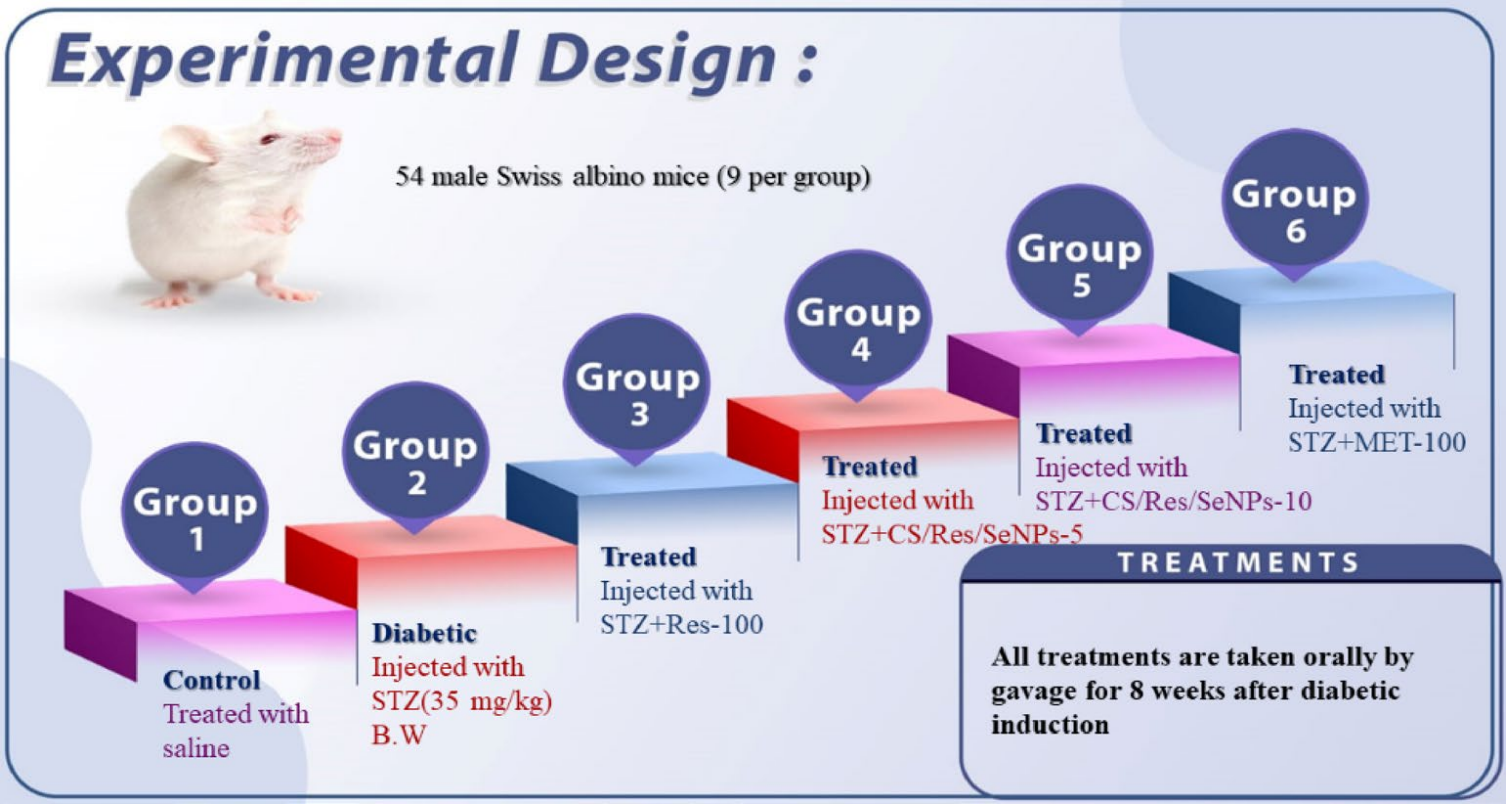
Experimental design and group classification.

### Induction of type 2 diabetes mellitus

Animal HFD dietary supplementation with 3% fat, 54% carbohydrate, 26% protein, and 17% vitamins and minerals was given to mice for 12 weeks as a means for T2DM induction. Elevated serum blood glucose, total cholesterol (TC), and triglycerides (TG) were indicators of hypercholesterolemia and hypertriglyceridemia in the pre-diabetic stage. After 12 weeks of HFD, mice that had fasted overnight for 16 h were given a single i/p injection of 35 mg/kg BW STZ (Sigma-Aldrich, USA), dissolved in 0.1 M citrate buffer pH 4.5. Diabetes has been confirmed to have developed 72 h after receiving an STZ injection. For the experiment, animals with blood glucose levels between 300 and 450 mg/dL will be used. The blood glucose levels were determined using the CareSens S Fit glucometer.

### Experimental design and animal treatment

Mice were divided randomly into six groups as shown in Fig. 1. Mice were grouped as follows:

The control group (*n* = 9): mice obtained normal saline daily by intragastric tube.

T2DM-induced group (*n* = 9): After a 12-week HFD administration followed by a 16-hour fasting, the mice received a single i/p injection of 35 mg/kg BW of STZ. STZ was dissolved in a freshly prepared citrate buffer (0.1 M, pH 4.5). Animals with fasting blood glucose between 300 and 450 mg/dL, three days after STZ administration, were considered diabetic and they received treatment similar to that of normal mice.

T2DM + Res-100 (*n* = 9): Mice were given 100 mg/kg of Res orally every day for eight weeks after being induced by HFD/STZ.

T2DM + CS/Res/Se-NPs-5 (*n* = 9): Mice were induced with HFD/STZ and then administered oral Res-loaded CS nanoparticles at a dose of 5 mg/kg once a day for 8 weeks.

T2DM + CS/Res/Se-NPs-10 (*n* = 9): Mice were given oral Res-loaded CS nanoparticles at a concentration of 10 mg/kg once daily for eight weeks after being induced by HFD/STZ.

T2DM + Met-100 (*n* = 9): Mice were induced by HFD/STZ and then treated orally with Met 100 mg/kg daily for 8 weeks. Each group was given their prescribed dosage orally for eight weeks. Every day at the same time, following breakfast, mice received a dose of each treatment. The Res solution was made by dissolving the prescribed dosage of Res-100, CS/Res/Se-NPs-5, CS/Res/Se-NPs-10, and Met-100 in a normal saline solution based on each mouse’s body weight, and the mice then took it orally.

The mice were anesthetized with sodium pentobarbital (100 mg/kg) after fasting all night. Following the cessation of the righting reflex, the animals were euthanized via heart puncture, and blood samples were collected and allowed to clot for 30 min at room temperature. Certain liver tissues were immediately removed and stored at (− 80℃) (Revco, USA) for RNA isolation. The remaining liver tissues were collected on ice for the remaining tests. For histological analysis, a portion of the liver was preserved in 10% formalin. Mouse sera were stored at −20℃ for biochemical analysis.

### Body weight Estimation

To assess body weight changes, the body weight of every mouse in each group was measured once a week during the entire experimental study (Table S1).

### Evaluation of serum glucose, insulin, and HOMA-IR

Quantitative determination of glucose was performed using a specific kit purchased from Spectrum Diagnostics (Egypt) as directed by the manufacturer’s guidelines. Mice-specific kits from MyBioSource, USA, were used to measure insulin quantitatively in compliance with the ELISA technique, according to the manufacturer’s protocols. In addition, the homeostatic model assessment of insulin resistance (HOMA-IR) was determined using the following formula: $\:\text{H}\text{O}\text{M}\text{A}-\text{I}\text{R}\:=\frac{\text{G}\text{l}\text{u}\text{c}\text{o}\text{s}\text{e}\:\left(\text{m}\text{g}/\text{d}\text{L}\right)\times\:\text{I}\text{n}\text{s}\text{u}\text{l}\text{i}\text{n}({\upmu\:}\text{I}\text{U}/\text{m}\text{L})}{405}$

### Lipid, liver, and kidney profile assessment protocols

Serum levels of TC, high-density lipoprotein cholesterol (HDL-c), low-density lipoprotein cholesterol (LDL-c), TG, alanine transaminase (ALT), aspartate transaminase (AST), alkaline phosphatase (ALP), albumin, total protein, urea and creatinine were measured commercially using available kits specific for each parameter (Spectrum Diagnostics, Egypt) according to manufacturer’s instructions.

### Antioxidant and oxidative stress biomarkers

Using a homogenizer, the isolated liver tissue was then mixed with a lysis buffer including a protease inhibitor (150 mM NaCl, 1% Triton X-100, 10 mM Tris, pH 7.4) and centrifuged at 10,000× g for 15 min at 4 °C. The Lowry method was used to measure the total protein content^18^. Superoxide dismutase (SOD), reduced glutathione, catalase (CAT), GPx, Glutathione reductase (GR), Glutathione-S-transferase (GST) activities in the liver were estimated following the previously outlined method^19–24^. Lipid peroxidation was assessed by detecting thiobarbituric acid reactive substances (TBARS), which are indicators of oxidative stress in the liver. Additionally, nitric oxide (NO) was measured as previously described^25,26^.

### Hepatic pro-inflammatory and anti-inflammatory cytokines

Liver homogenate samples were tested for Interleukin (IL)−18 (MyBioSource, MBS265871), Inducible nitric oxide synthase (iNOS) (MyBioSource, MBS261100), Tumor necrosis factor α (TNF-α) (MyBioSource, MBS2500421), IL-6 (MyBioSource, MBS730957), IL-1β (MyBioSource, MBS175967), and IL-10 (MyBioSource, MBS2021945) using a mouse-specific ELISA kit according to the manufacturer’s protocols. Using an ELISA plate reader (BMGLABTECH^®^FLUOStar Omega, Germany), the absorbance was measured at 450 nm.

### Apoptotic markers gene expression using quantitative real-timepolymerase chain reaction (qRT-PCR)

The liver tissues’ total RNA was isolated using the RNeasy Mini Kit (Qiagen). Using a commercial first-strand cDNA synthesis kit, 1 µg of total RNA was utilized to create cDNA following the SensiFAST cDNA Synthesis kit’s manufacturer’s instructions. The cDNA samples were run in triplicate for a quantitative real-time PCR analysis, as well as the expression level of genes associated with internal regulation, and Glyceraldehyde-3-phosphate dehydrogenase (GAPDH) was determined. First, denaturation was carried out at 95 °C for 10 min. This was followed by 40 cycles of 10 s at 95 °C, 10 s at 60 °C, and 20 s at 72 °C. The threshold cycle values were utilized to estimate the RNA concentration. The comparative threshold cycle approach (2^−ΔΔCq^) was employed to calculate the levels of gene expression, with ΔCq signifying the variations in Cq values between the target gene and GAPDH^27^. The Primer sequences (Sigma-Aldrich, USA) were as follows in Table 1:

**Table 1:** Primer sequence for RT-PCR analysis.

Gene	Sequences (5′ to 3′)
Bax	F- 5′-GCAAACTGGTGCTCAAGG-3′ R- 5′-TCCCGAAGTAGGAAAGGAG-3′
BCL2	F- 5′-GGGATACTGGAGATGAAGACT-3′ R- 5′-CCACCGAACTCAAAGAAGG-3′
Caspase-3	F- 5′-TTAATAAAGGTATCGATGGAGAACACT-3′ R- 5′- TTAGTGATAAAAATGAGTTCTTTTGTGAG-3′
Caspase-8	F- 5′-CTGCTGGGGATGGCCACTGTG-3′ R- 5′-TCGCCTCGAGGACATCGCTCTC-3′
GAPDH	F- 5′-GTATTGGGCGCCTGGTCACC-3′ R- 5′-CGCTCCTGGAAGATGGTGATGG-3′

### Immunohistochemical examination

NF-kB and iNOS protein expression levels were assessed using the immunohistochemistry (IHC) technique. Liver tissue sections were fixed by immersion in 10% formalin buffered solution (pH 7.4) for 24 h. Following fixation, the sections were washed twice with phosphate-buffered saline (PBS). The sections were then washed an additional two times with PBS containing 0.1% saponin (PBS-S; Sigma). Endogenous peroxidase activity was quenched by incubating the sections in a solution containing 1% hydrogen peroxide, 0.3 M sodium azide (Sigma), and 0.1% saponin. After this blocking step, the sections were rinsed again with PBS-S before subsequent immunostaining procedures. Microscopic glass slides coated with poly-L-lysine were used to mount tissue samples embedded in paraffin. Tissues were rehydrated in sequentially graduated ethanol, and after sections were submerged in xylene to deparaffinize them. Non-specific binding sites were blocked with Novocastra Protein Block (RE7102, Leica Biosystems, IL, USA) for an hour. Primary antibodies targeting NF-κB (MyBioSource, Cat. No. MBS633051) and iNOS (MyBioSource, Cat. No. MBS9601541) were applied to the tissue sections and incubated overnight at 4 °C to allow for antigen-antibody binding. Following extensive washing, the tissue sections were incubated for 2 h at room temperature with a biotinylated secondary antibody to detect bound primary antibodies. To visualize the antigen-antibody complexes, sections were then treated with the avidin-biotin-peroxidase complex (ABC kit, Vector Laboratories, Newark, CA, USA) for 1 h. Finally, the slides were incubated for 6–10 min with colored diaminobenzidine substrate (DAB) (Sigma-Aldrich, St. Louis, MO, USA) until a brown precipitate formed and were subsequently counterstained with haematoxylin. The stained sections were examined under a light microscope, and images were captured using a digital camera (Nikon Corporation Co., Ltd., Japan).

### Histological investigations

The liver tissue was dehydrated in an ascending graded series of alcohol, cleaned triplicate in xylene for 1 h, impregnated in melted paraffin and wax, and then an oven for 1 h. After solidifying, rotatory microtomes were used to cut 5 μm-thick sections, which were then placed on glass slides, stained with hematoxylin and eosin (H&E), and examined for any histopathological changes.

### Molecular docking

Using computer-based chemistry approaches, the probable inhibitive activities of Res against specific sites have been investigated. Initially, proteins (proteins ID: 3QKK, 4BFR, 2OJ9, 3ML9, and 5BTR) were downloaded from the protein data bank. After preparing all proteins and Res, an MMFF94 force field was used to minimize energy^28,29^. At first, water molecules were removed, and unnecessary molecules were neglected from the protein complexes. Then, the disorders and unfilled valence atoms were corrected. The protein structures’ energies were minimized and saved as PDBQT files. The 2D structure of Res was drawn using Chem-Bio Draw Ultra16.0, saved as an SDF file, preparation was established and saved as PDBQT file, and the prepared ligand was docked against the previous targets using Autodock Vina 1.5.7 software. The docking was conducted by a rigid technique; in this method, the receptor was held rigid while the ligands were allowed to be flexible. During the docking refinement, each molecule was allowed to generate twenty different poses. The docking scores (affinity energy) of the best-fitted poses with the active sites were recorded, and 3D/2D figures were generated using Discovery Studio 2024 visualizer.

### Molecular dynamics (MD) simulation studies

MD simulations were performed for 100 ns to assess the stability of the Res with the best docking score in the silent information regulator sirtuin 1 (SIRT-1) and AKT active sites. The obtained Root Mean Square Deviation (RMSDs) of the ligands and complexes in relation to their initial positions inside the active site were investigated and reported. Additionally, boundary chemical interactions were thoroughly investigated and evaluated. Finally, during the simulation trajectory, each complex’s molecular mechanics with generalized born surface area (MM-GBSA) free binding energy was estimated.

### Statistical analysis

Values are expressed as mean ± standard error of the mean (SEM) at *p* < 0.05. The statistical significance was determined by one-way analysis of variance (ANOVA) using the Primer of Biostatistics (Version 5) software program, followed by Tukey’s post hoc test for multiple comparisons. Statistical significance was set at *p* < 0.05, which means that the difference in the groups was deemed statistically significant when *p* < 0.05, utilizing the GraphPad Prism program v.8.4.3 for graphing.

## Results

### Swiss target prediction

**Fig. 2:**
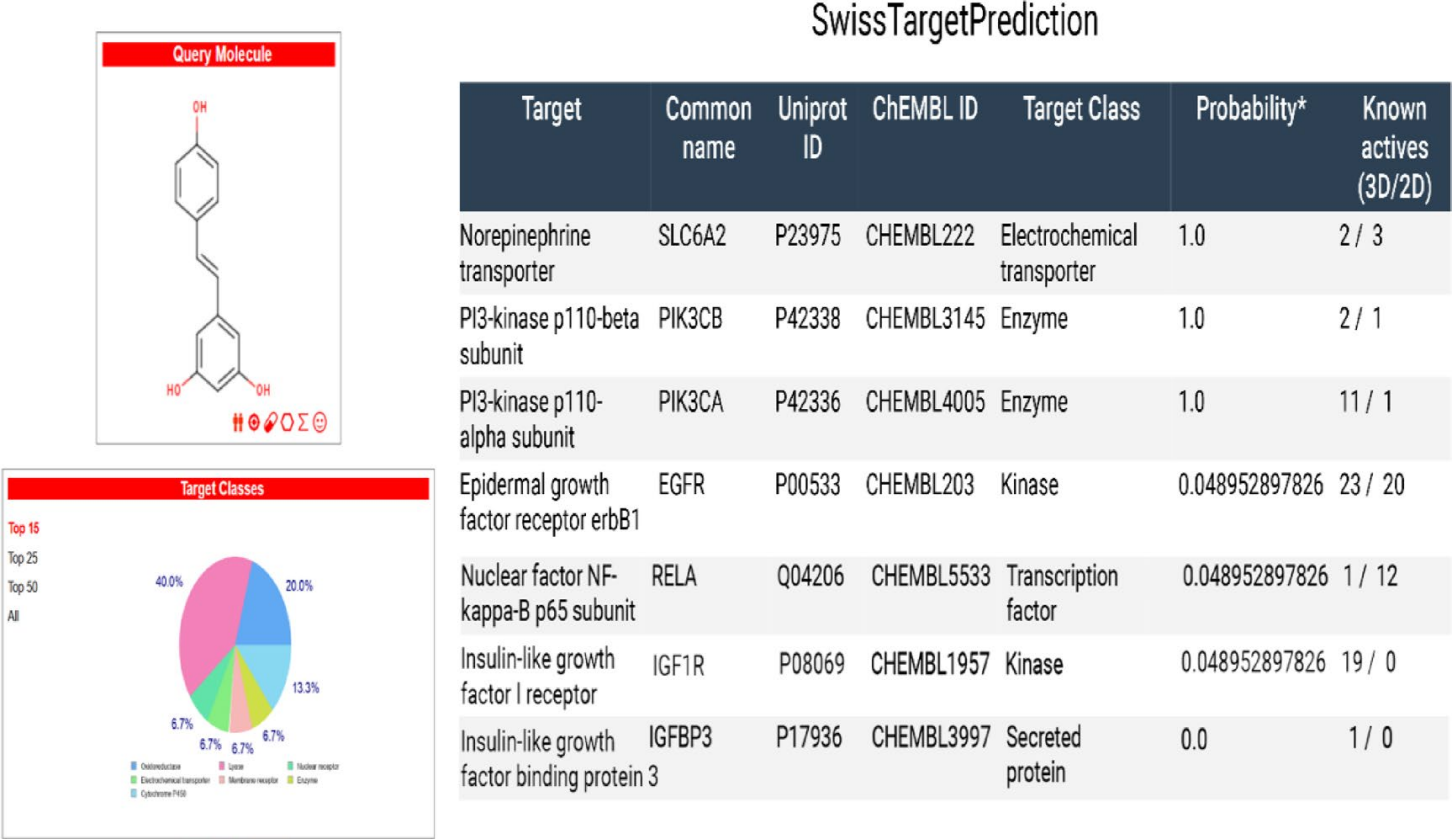
Human target prediction. Swiss Target Prediction indicates that Res possesses a high probability of targeting IGF1R, IGFBP3, SLC6A2, PIK3CB, PIK3CA, EGFR, and NF-κB p65 subunit based on combined 2D and 3D structural similarity analyses.

The Swiss Target Prediction web server predicts molecular targets by comparing a query molecule, such as Res, with a database of known active compounds using similarity measures in both 2D and 3D space. Figure 2 depicts the Swiss Target prediction results, which include a list of Res’ expected targets. The fifth column shows the projected probability that a protein will be a Res target. The sixth column (3D/2D) shows the number of ligands that have structural similarities to Res.

### GO function enrichment analysis

**Fig. 3:**
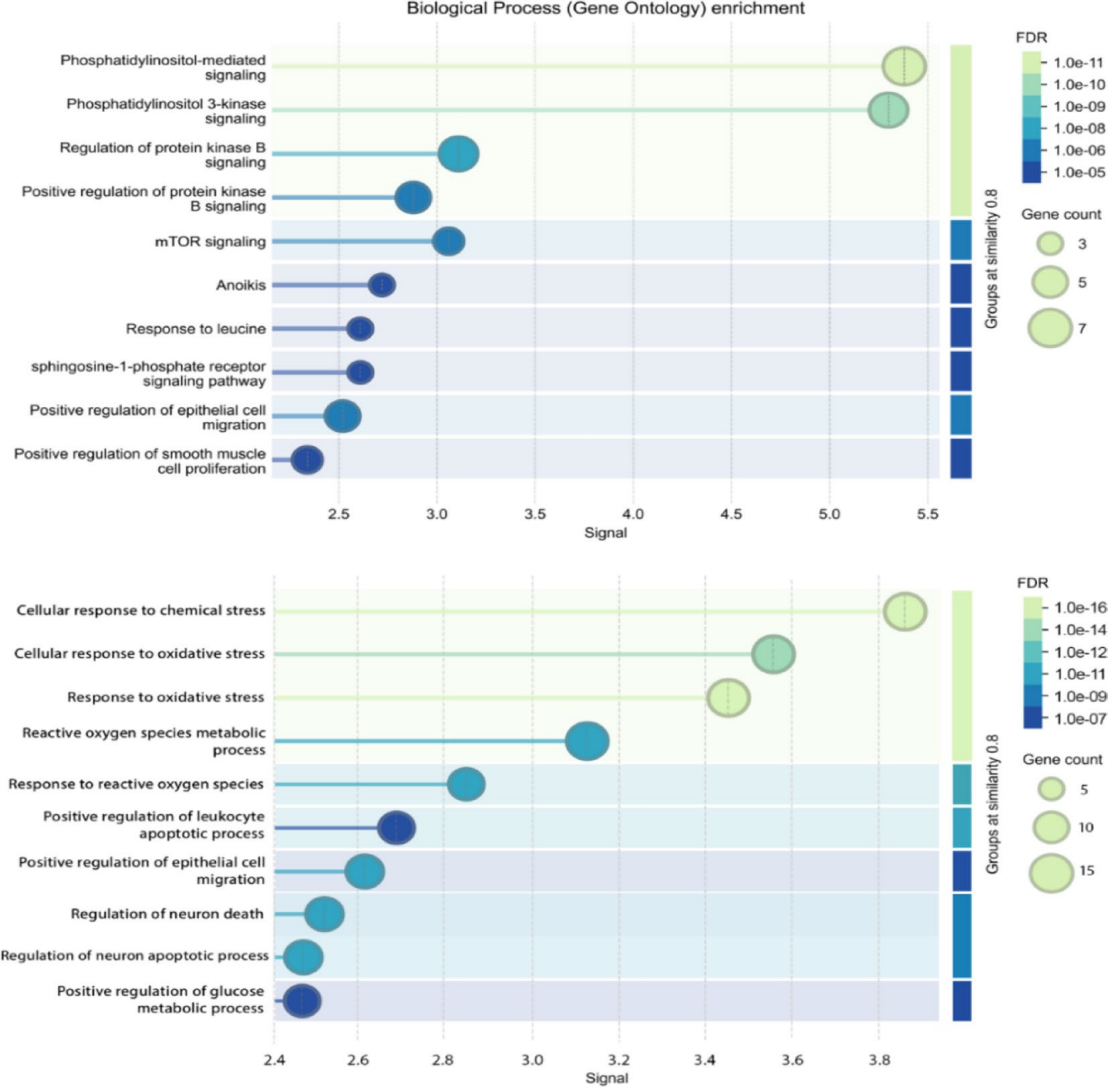
GO enrichment analysis of target proteins.

The GO enrichment analysis results of key molecular targets of Res are shown in Fig. 3, *p* < 0.05. The GO enrichment results revealed that PI3K/AKT/mTOR signaling pathway might play a role in the inhibitory effects of Res on DM and its regulation of multiple biological processes. These biological processes include regulation of the glucose metabolic process, regulation of neuron apoptotic process, cellular response to oxidative stress, positive regulation of PI3K signaling, response to hypoxia, leukocyte migration, positive regulation of AKT signaling, and positive regulation of epithelial cell migration.

### PPI network of target genes

**Fig. 4:**
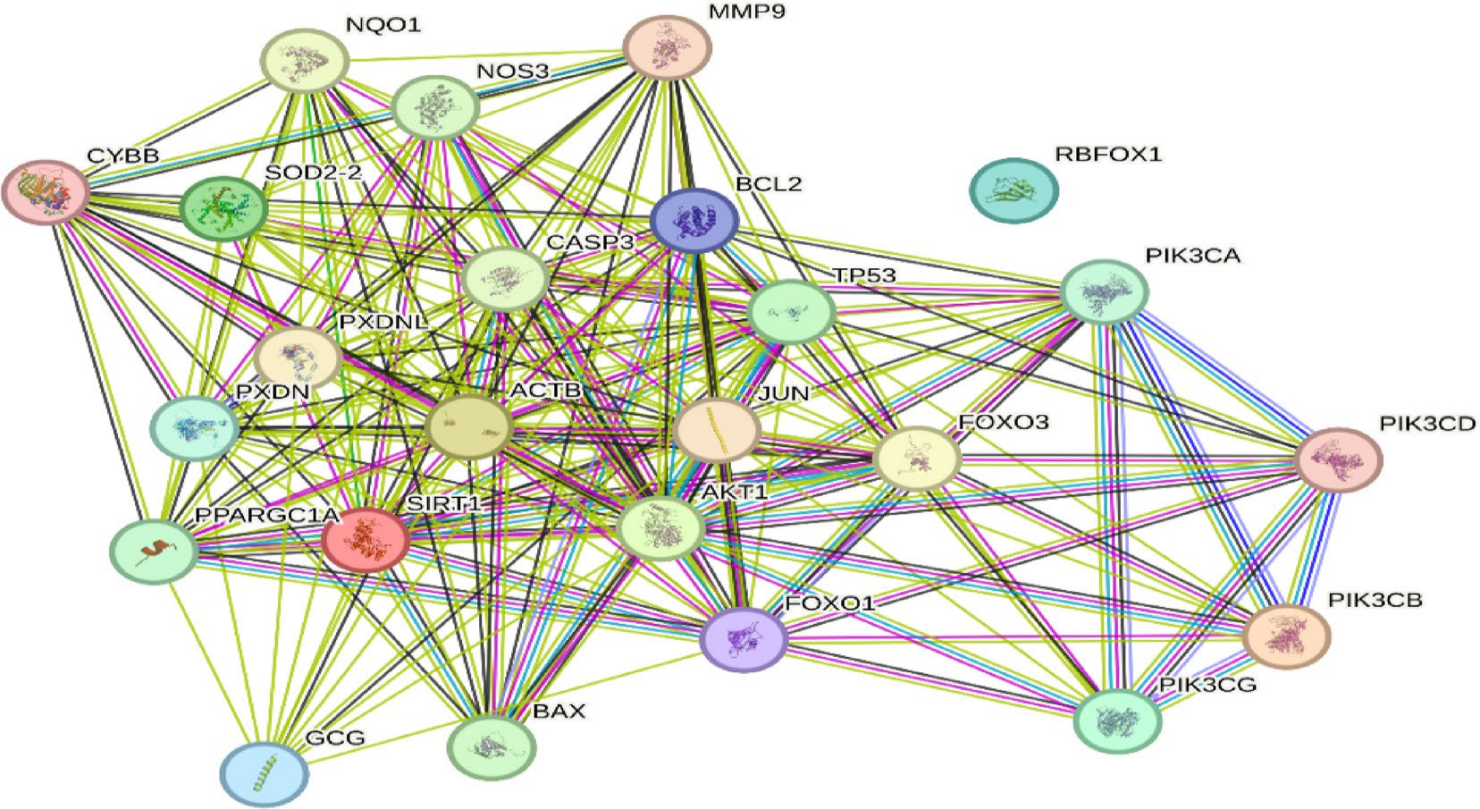
PPI network, generated using the STRING database. This network illustrates key regulatory proteins targeted by res, which are involved in critical cellular pathways including insulin signaling, apoptosis, oxidative stress, and inflammation.

This STRING-derived PPI network reveals a tightly connected regulatory framework involving key molecular players modulated by Res in oxidative stress response, apoptosis, insulin signaling, and inflammatory pathways, as shown in Fig. 4. The software produces score information for each pair of proteins. The higher the score, the more confident the target protein’s interactions were. Thus, the potential targets modulated by Res were imported into STRING tool to acquire protein-protein interactions. We selected a high confidence score > 0.7 with the species restricted to “Homo sapiens”. Then, target genes with high degree were selected as the hub genes of key targets for Res. Central hub proteins like AKT1, tumor protein p53 (TP53), caspase 3 (CASP3), and SIRT1 serve as key regulators that integrate upstream oxidative signals with downstream decisions related to cell fate and metabolic regulation. The presence of PI3K family proteins and Forkhead box O (FOXO) transcription factors highlights the modulation of insulin/insulin-like growth factor (IGF-1) signaling and oxidative stress resistance pathways. Importantly, interactions between SIRT1, peroxisome proliferator-activated receptor gamma coactivator 1 (PPARGC1A), and FOXO1/FOXO3 indicate a strong mitochondrial and antioxidant component, aligning with their roles in metabolic disease and aging. On the apoptotic front, the B-cell lymphoma 2 (BCL2)/Bcl-2-associated X protein-(BAX)/CASP3 axis illustrates the intricate balance between pro- and anti-apoptotic signals, which are critical in cellular response to oxidative damage. The involvement of cytochrome b-245 beta chain (CYBB) and SOD2 further confirms the network’s relevance to redox regulation.

### Characterization of Res-loaded CS nanoparticles (CS/Res/SeNPs)

**Fig. 5:**
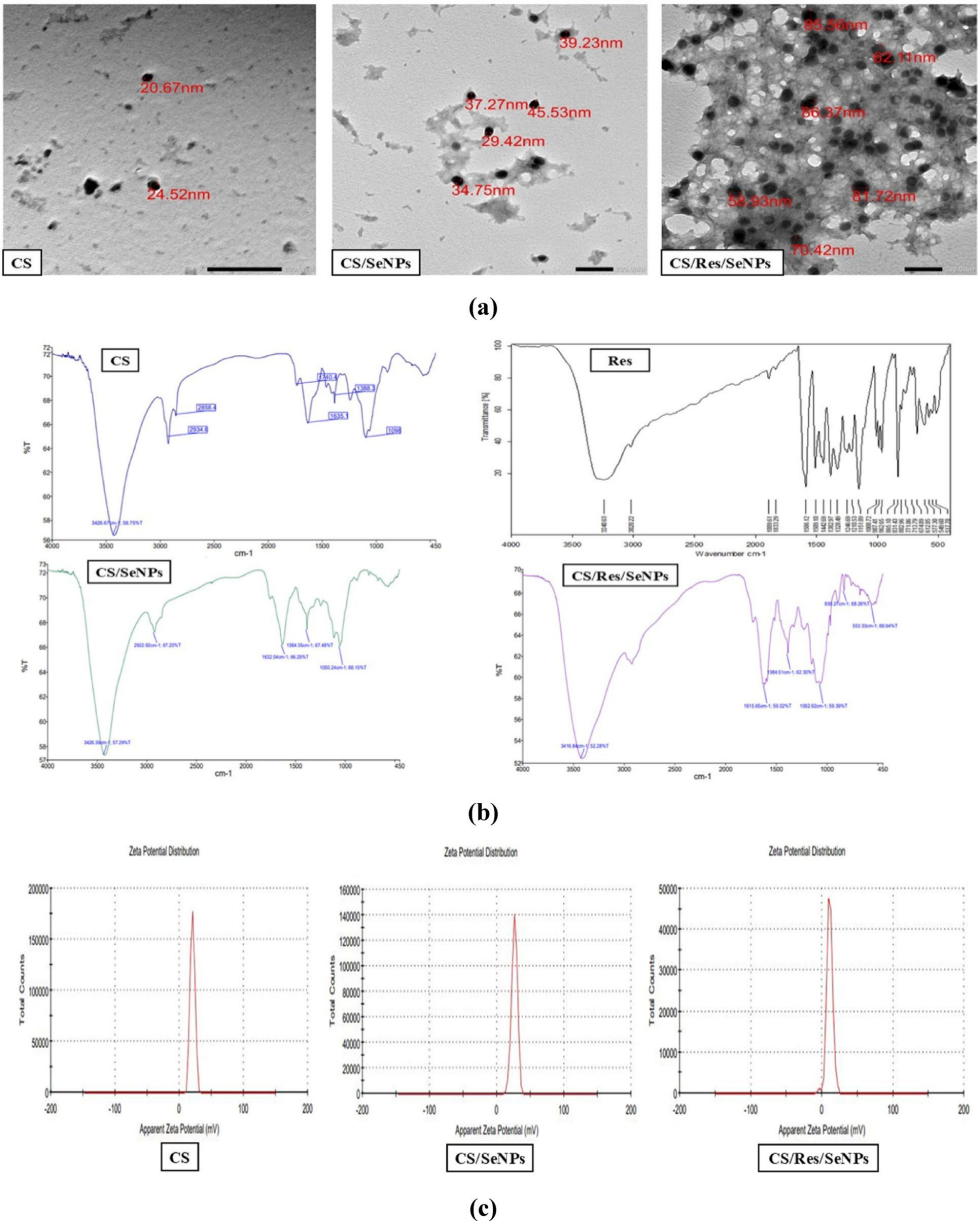
Characterization of the prepared nanoparticles. **(a)** TEM of free CS; CS/SeNPs and CS/Res/SeNPs, **(b)** FTIR pattern of CS; Res; CS/SeNPs; CS/Res/SeNPs, **(c)** Zeta-potential of CS; CS/SeNPs and CS/Res/SeNPs.

The morphological characteristics of our prepared nanoparticles were evaluated using TEM. The TEM analysis images revealed that free CS nanoparticles were spherical and had a diameter between 20 and 24 nm, whereas CS/SeNPs appeared with a diameter between 34 and 45 nm. Moreover, CS/Res/SeNPs appeared spherical, with the majority of the nanoparticles having a diameter of 59–86 nm (Fig. 5a)**.**

Distinctive absorption spectrum from CS’s functional groups may be seen in its FTIR spectra (Fig. 5b)**.** The region in which the spectra were collected was between 450 and 4000 cm^−1^. For example, the absorption spectrum bands at 2858.4 cm^−1^ and 2934.6 cm^−1^ are driven by the C–H bond stretch vibrations that are present in this polymer. The produced CS’s -OH bond stretching vibrations were detected at 3426.67 cm^−1^. The C = O’s stretching vibrations were then visible at a wavelength of 1740.4 cm^−1^. The absorption peaks around 1635.1–1388.3 cm^−1^ were associated with the existing amide I absorption band’s C = O stretching and OH bending, respectively. Furthermore, observed bending vibrations of the -C-O-C- groups, which are characteristic of polysaccharides, are responsible for the band at 1098 cm^−1^, having a comparatively high intensity relative to the others. Figure 5b shows the FTIR spectra of Res. Res demonstrated specific alcoholic/phenolic OH vibrational peaks at 3240.63 and 3020.22 cm^−1^. Res’s spectra revealed distinctive bands at 1889.61 and 1833.29 cm^−1^ that correspond to vibration in the benzene ring skeleton or stretching C = C and C–C groups. Res showed specific C–O stretching at 1246.69 and 1210.53 cm^−1^. The trans-Res structure is represented by a band at 987.41 and 962.65 cm^−1^ because of existing C-H stretching. Additionally, an alkyl bending vibrational peak was observed at 865.10 cm^−1^. At 831.43 and 802.96 cm^−1^, the arene conjugated to the olefinic group exhibits its C–H vibration band. The structure of the CS/SeNPs was investigated using FTIR spectroscopy to validate the chemical interactions between Se and CS (Fig. 5b). The bending oscillations of C–H and C–C interactions may be responsible for the characteristic peak that CS/SeNPs exhibited at 2922.92 cm^−1^. Furthermore, the vibrational bending for N–H is represented by the peak at 1632.54 cm^−1^, whereas the vibrational stretching for C–O as well as bends of O–H have been correlated to the peaks at 1384.35 and 1050.24 cm^−1^, respectively. The FTIR spectra of CS/Res/SeNPs (Fig. 5b) show three prominent absorption peaks at 1615.65, 1062.92, and 1384.51 cm^−1^, which have been correlated with aromatic C = C stretching, olefinic C–C bending, as well as Carbon–Carbon bending of Res, respectively. At 830.27 cm^−1^, Res showed distinctive bands with a strong aromatic ring’s C-H bend. As shown in Fig. 5c, zeta potential measurements of the prepared nanoparticles, CS, CS/SeNPs, and CS/Res/SeNPs indicate the extent of the surface charge of those nanoparticles in each sample. Free CS had a zeta potential of + 20.6 mV. However, the zeta potential of CS/SeNPs was + 26.1 mV. In contrast, the zeta potential values for CS/Res/SeNPs were moved from + 20 to + 10.9 mV.

### Effect of prepared nanoparticles on body weight changes

**Fig. 6:**
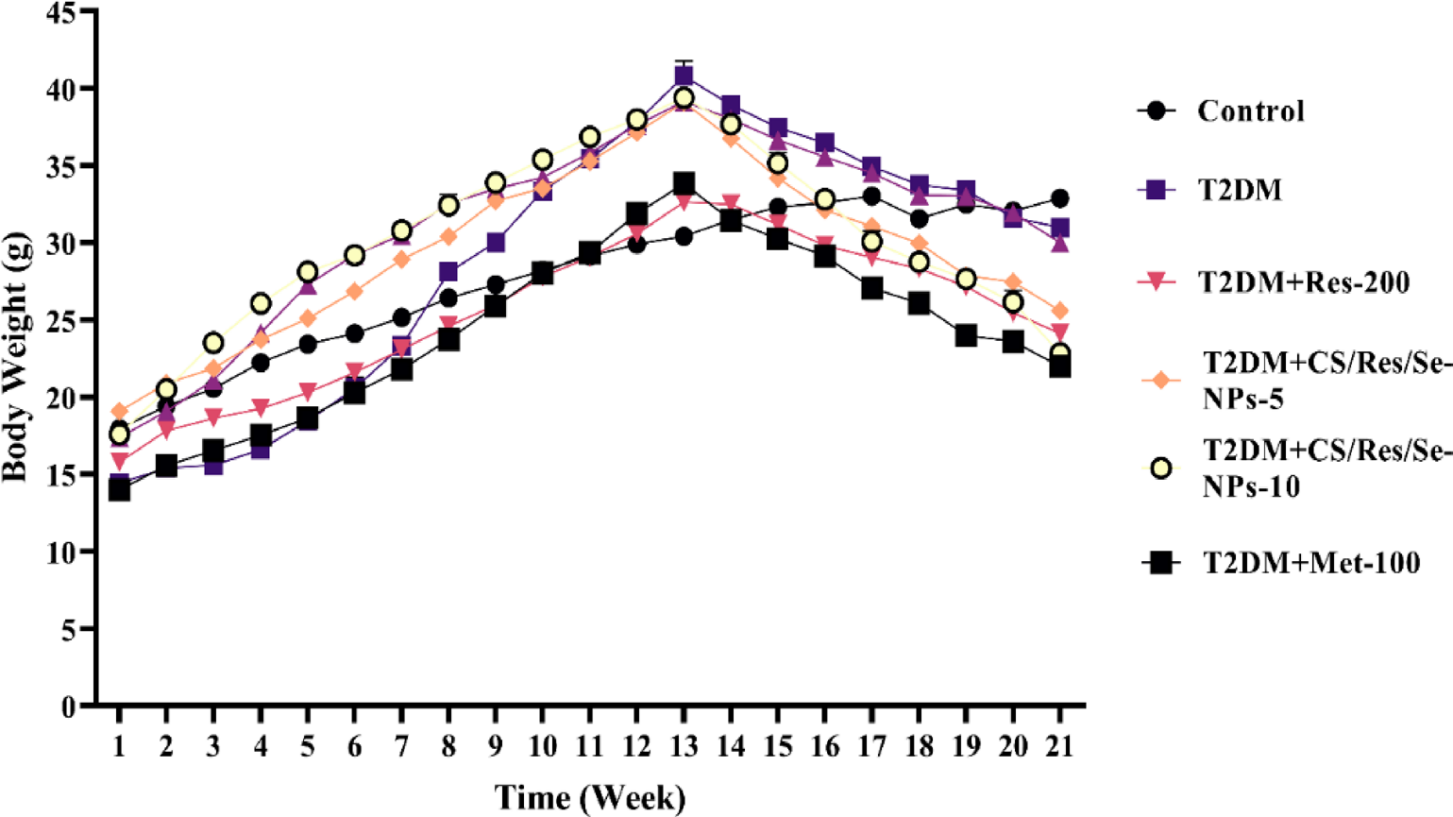
Changes in body weights during the experiment in all studied groups. Data are means ± SEM (*n* = 6); statistical significance was set at *p* < 0.05, Tukey.

The average body weight records for all different groups were the same at the onset of our experiment. The control group demonstrated a steady and progressive rise in body weight. Following 12 weeks of HFD delivery, the mice’s body weight increased. In contrast, a constant decrease in body weight was noted during the experimental period after STZ induction. Notably, the administration of CS/Res/Se-NPs-5 or CS/Res/Se-NPs-10, as well as standard Res-100, resulted in body weight loss during the entire course of the treatment, with an observable body weight loss in the group treated with Met-100 (Fig. 6).

### CS/Res/Se-NPs administration improves serum diabetic profile

As noted, typical clinical signs of T2DM-induced mice include observed IR as well as disturbance in glucose metabolism. The model group developed IR as a result of twelve weeks of HFD feeding and STZ injection, leading to a significant increase in fasting blood glucose (FBG) levels, which were 5-fold higher compared to control mice. Additionally, the diabetic model mice group exhibited a substantial rise in serum insulin levels in correlation with the elevated FBG levels compared to control mice (*p* < 0.05, Table 2). In contrast, treatment with CS/Res/Se-NPs-5 or CS/Res/Se-NPs-10, as well as standard Res-100 or Met-100, significantly reduced FBG levels (3.5, 4, 3.7, and 3.1-fold decrease, respectively) and insulin concentrations compared to the T2DM-induced mice (*p* < 0.05). These results suggested that CS/Res/Se-NPs and Met had positive impacts on reducing insulin and glucose levels. Besides, T2DM-induced mice showed a significant elevation in IR relative to control mice, as confirmed by high HOMA-IR. Notably, CS/Res/Se-NPs-5 or CS/Res/Se-NPs-10, as well as standard Res-100 or Met-100 administration, significantly reduced HOMA-IR levels relative to the T2DM-induced mice, reaching the control ranges (26, 38.8, 26, 7.5-fold decrease, respectively, Table 2).

**Table 2:** Change in serum diabetic profile levels in all studied groups.

Groups	FBS (mg/dL)	Insulin (mIU/mL)	HOMA-IR
Control	81.33 ± 5.93 ^e^	2.67 ± 0.09 ^c^	0.53 ± 0.04 ^d^
T2DM	427.33 ± 3.71 ^a^	22.07 ± 0.48 ^a^	23.27 ± 0.30 ^a^
T2DM + Res-100	114.33 ± 1.76 ^c, d^	3.20 ± 0.04 ^c^	0.90 ± 0.01 ^d^
T2DM + CS/Res/Se-NPs-5	123.67 ± 3.71 ^b, c^	2.91 ± 0.04 ^c^	0.88 ± 0.02 ^d^
T2DM + CS/Res/Se-NPs-10	97.00 ± 3.79 ^d, e^	2.57 ± 0.04 ^c^	0.61 ± 0.03 ^d^
T2DM + Met-100	135.67 ± 2.60 ^b^	9.19 ± 0.06 ^b^	3.07 ± 0.07 ^c^

The values are presented as mean ± SEM (*n* = 6), the highest data value is represented by the letter (a), and the smallest data value is represented by the letter (e). The means for the same parameter with different letters in each column are substantially different (Tukey, *p* < 0.05).

### CS/Res/Se-NPs administration improves serum liver profile

T2DM-induced mice exhibited increased hepatic enzyme activities ALT, AST, and ALP, along with decreased levels of liver function-related proteins albumin and total protein, relative to control mice. Treatment with CS/Res/Se-NPs at both 5 mg/kg and 10 mg/kg, as well as standard Res-100, resulted in a significant reduction in hepatic enzyme levels (*p* < 0.05) relative to the untreated diabetic group. The administration of CS/Res/Se-NPs (5 or 10 mg/Kg) resulted in a significant increase in serum levels of albumin and total protein; Met treatment produced comparable effects. Notably, administration of current treatment options resulted in a normalization of serum liver profile, with values comparable to control values **(**Table 3**)**.

**Table 3:** Change in serum liver profile in all studied groups.

Groups	ALT (U/L)	AST (U/L)	ALP (U/L)	Albumin (g/dL)	Total protein (g/dL)
Control	38.47 ± 0.84 ^b^	99.58 ± 1.83 ^b^	91.73 ± 0.31 ^b^	3.63 ± 0.18 ^a^	7.03 ± 0.30 ^a, b^
T2DM	181.14 ± 2.05 ^a^	242.13 ± 2.67 ^a^	191.53 ± 4.51 ^a^	2.23 ± 0.09 ^b^	4.43 ± 0.09 ^d^
T2DM + Res-100	32.70 ± 2.17 ^b^	86.47 ± 1.30 ^c^	71.47 ± 2.98 ^c^	4.37 ± 0.18 ^a^	6.33 ± 0.13 ^c^
T2DM + CS/Res/Se-NPs-5	33.57 ± 0.12 ^b^	94.63 ± 2.06 ^b, c^	72.70 ± 1.17 ^c^	4.07 ± 0.09 ^a^	7.30 ± 0.06 ^a^
T2DM + CS/Res/Se-NPs-10	30.97 ± 1.76 ^b^	99.70 ± 2.43 ^b^	94.93 ± 0.29 ^b^	3.57 ± 0.42 ^a^	6.63 ± 0.09 ^a, b,c^
T2DM + Met-100	36.13 ± 1.62 ^b^	92.23 ± 2.54 ^b, c^	72.23 ± 0.75 ^c^	3.43 ± 0.23 ^a^	6.40 ± 0.06 ^b, c^

Values are expressed as mean ± SEM (*n* = 6). Means for the same parameter with different letters in each column are significantly different (Tukey, *p* < 0.05). The biggest data value corresponds to the letter (a), while the smallest corresponds to the letter (d).

### CS/Res/Se-NPs administration enhances serum renal function

The current findings demonstrated a notable elevation in serum creatinine and urea concentrations in T2DM-induced mice, compared to control mice (*p* < 0.05). Treatment with doses (5 or 10 mg/kg) of CS/Res/Se-NPs, as well as regular Res-100 or Met-100, contributed to a substantial decrease in both creatinine and urea levels relative to the untreated mice, reaching normal range values (*p* < 0.05, Table 4).

**Table 4:** Change in serum renal profile in all studied groups.

Groups	Creatinine (mg/dL)	Urea (mg/dL)
Control	0.41 ± 0.01 ^e^	10.00 ± 0.58 ^c^
T2DM	1.88 ± 0.05 ^a^	46.67 ± 3.33 ^a^
T2DM + Res-100	0.68 ± 0.01 ^c, d^	16.33 ± 0.33 ^b, c^
T2DM + CS/Res/Se-NPs-5	0.80 ± 0.07 ^b, c^	18.00 ± 0.58 ^b, c^
T2DM + CS/Res/Se-NPs-10	0.55 ± 0.01 ^d, e^	20.00 ± 2.52 ^b^
T2DM + Met-100	0.90 ± 0.05 ^b^	11.33 ± 0.33 ^c^

Values are expressed as mean ± SEM (*n* = 6), means for the same parameter with different letters in each column are significantly different (Tukey, *p* < 0.05), the highest data value takes the letter (a) and the smallest data value takes the letter (e).

### CS/Res/Se-NPs administration improves serum lipid profile

T2DM-induced mice exhibited a notable increase (*p* < 0.05) in circulating serum TG, TC, LDL, and a marked decline in HDL concentrations relative to the control animals. As illustrated in Table 5, diabetic mice that received CS/Res/Se-NPs-5 or CS/Res/Se-NPs-10, as well as standard Res-100 or Met-100, exhibited an anti-hyperlipidemic effect. Except for serum HDL levels, the lipid parameters of the treated mice were significantly reduced (*p* < 0.05) relative to those of the untreated animals. Notably, treated mice demonstrated a considerable rise in serum HDL levels relative to the T2DM-induced group.

**Table 5:** Change in serum lipid profile in all studied groups.

Groups	Cholesterol (mg/dL)	TG )mg/dL)	HDL )mg/dL)	LDL )mg/dL)
Control	98.33 ± 1.20 ^d^	71.00 ± 2.08 ^c^	71.00 ± 3.06 ^a^	56.00 ± 2.08 ^e^
T2DM	416.67 ± 23.33 ^a^	364.00 ± 19.16 ^a^	23.667 ± 1.76 ^d^	136.33 ± 1.76 ^a^
T2DM + Res-100	129.33 ± 4.81 ^c, d^	149.33 ± 2.40 ^b^	56.00 ± 3.00 ^b^	72.33 ± 1.86 ^c, d^
T2DM + CS/Res/Se-NPs-5	169.67 ± 10.53 ^b, c^	145.67 ± 2.19 ^b^	51.00 ± 1.00 ^b, c^	80.33 ± 1.20 ^b, c^
T2DM + CS/Res/Se-NPs-10	107.33 ± 2.19 ^d^	83.00 ± 2.52 ^c^	58.33 ± 2.19 ^b^	62.67 ± 1.45 ^d, e^
T2DM + Met-100	200.67 ± 4.70 ^b^	156.00 ± 2.65 ^b^	41.33 ± 0.88 ^c^	90.33 ± 4.48 ^b^

Values are expressed as mean ± SEM (*n* = 6), means for the same parameter with different letters in each column are significantly different (Tukey, *p* < 0.05), the highest data value takes the letter (a) and the smallest data value takes the letter (e).

### CS/Res/Se-NPs administration ameliorates hepatic oxidative stress

**Fig. 7:**
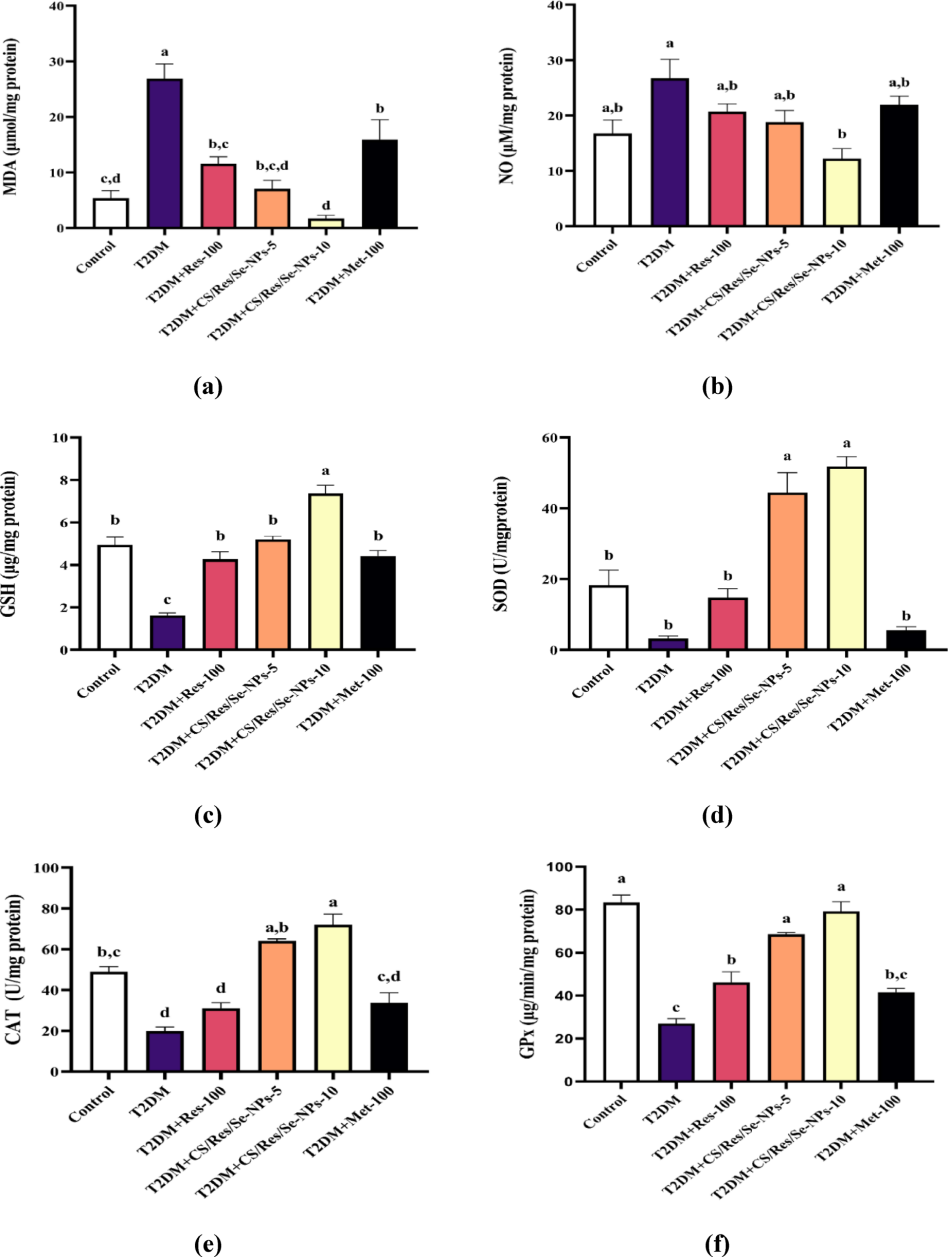

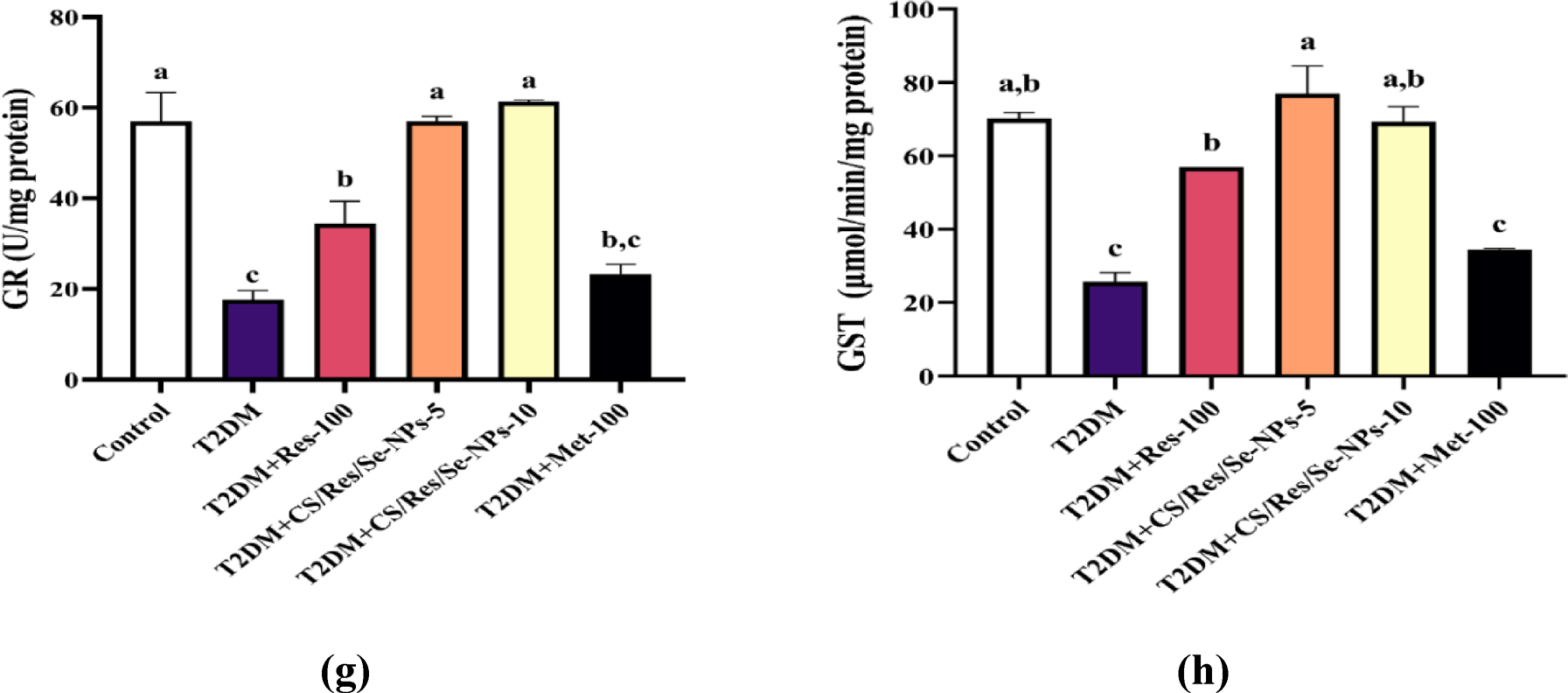
Changes in oxidative stress and antioxidants in all studied groups. (**a**) MDA concentration, (**b**) NO concentration, (**c**) GSH concentration, (**d**) SOD activity, (**e**) CAT activity, (**f**) GPx activity, (**g**) GR activity, and (**h**) GST activity. Data are means ± SEM (*n* = 6). Means with different letters in every bar indicate significant differences (Tukey, *p* < 0.05). The letter (a) is assigned to the highest data value, while the letter (d) is assigned to the smallest data value.

Our current results demonstrated that supplementation with HFD for 12 weeks, followed by STZ injection, led to a significant increase in hepatic malondialdehyde (MDA) and nitric oxide (NO) levels in T2DM-induced mice by approximately 5-fold and 1.6-fold, respectively, compared to control mice (*p* < 0.05; Fig. 7a&b). These increases in hepatic oxidative stress biomarkers were significantly reduced following the administration of CS/Res/Se-NPs-5 and/or CS/Res/Se-NPs-10 as well as standard Res-100 or Met-100, in comparison to T2DM-induced animals. On the other hand, the concentrations of GSH, a non-enzymatic antioxidant, and the levels of enzymatic antioxidants, including SOD, CAT, GPx, GR, and GST, showed a dramatic decrease (3.1, 5.7, 2.5, 3.1, 3.2, and 2.7-fold, respectively) in T2DM-induced group compared to control animals (*p* < 0.05; Fig. 7c-h). Interestingly, administration of CS/Res/Se-NPs-5 and/or CS/Res/Se-NPs-10 as well as standard Res-100 or Met-100 resulted in a remarkable restoration of GSH and SOD levels and a significant elevation of CAT, GPx, GR, and GST enzymatic activity compared to T2DM-induced mice (*p* < 0.05).

### CS/Res/Se-NPs administration inhibits hepatic cell apoptosis

**Fig. 8:**
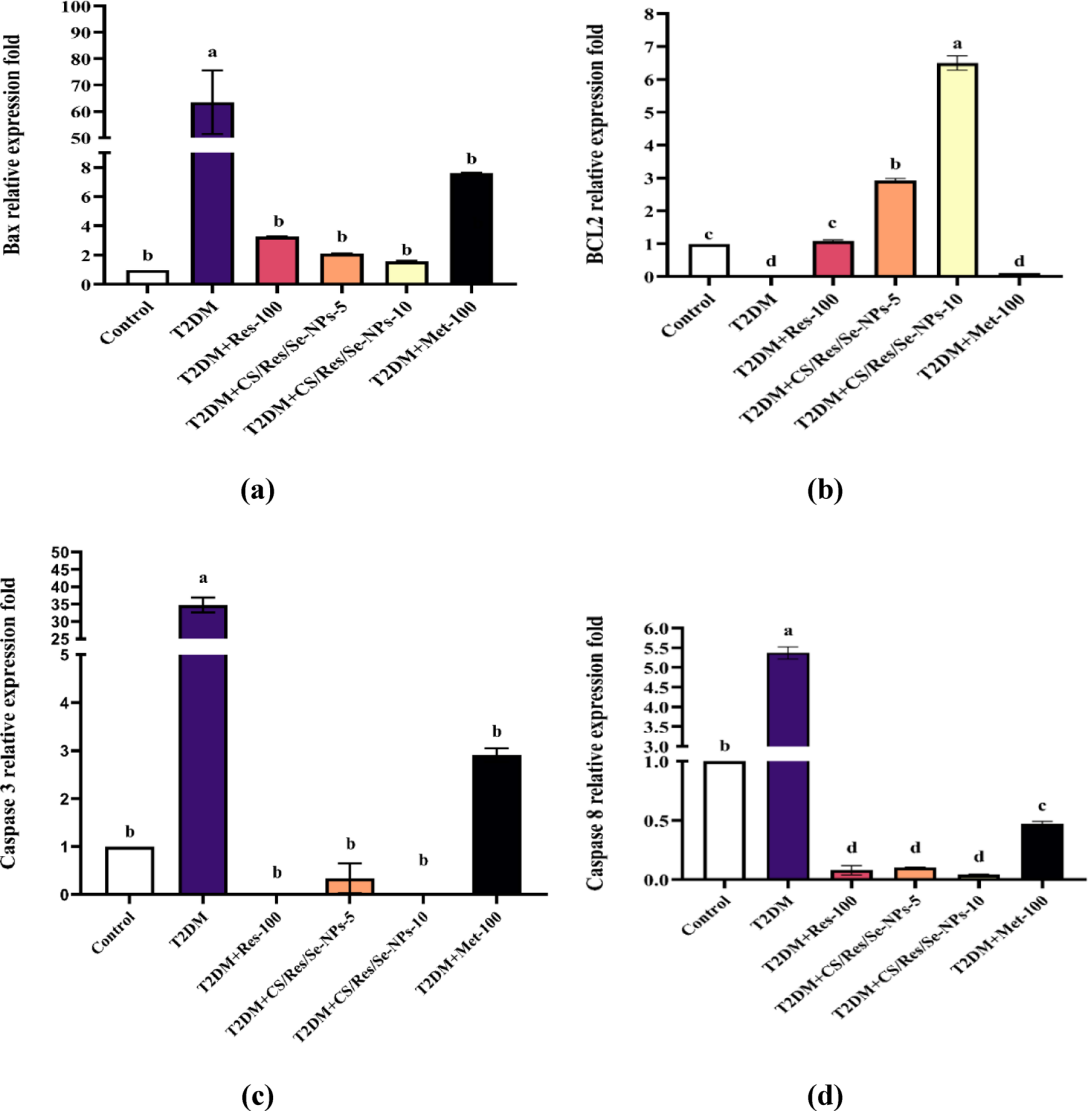
Gene expression profile of apoptotic markers in all studied groups. (**a**) Bax level, (**b**) BCL-2 level, (**c**) Caspase-3 level, and (**d**) Caspase-8 level. Data are means ± SEM (*n* = 6). Means with different letters in every bar indicate significant differences (Tukey, *p* < 0.05). The letter (a) is assigned to the highest data value, while the letter (d) is assigned to the smallest data value.

Under stressful conditions, the livers of T2DM-induced mice exhibited a marked reduction in Bcl-2 expression (*p* < 0.05). In contrast, the expression levels of Bax, Caspase-3, and Caspase-8 were significantly elevated compared to the control group. Notably, treatment with CS/Res/Se-NPs-5 or CS/Res/Se-NPs-10, as well as standard Res-100 or Met-100, led to a significant reduction in the expression of the pro-apoptotic proteins Bax, Caspase-3, and Caspase-8, along with an increase in Bcl-2 expression in the liver tissue of the mice (Fig. 8a-d, *p* < 0.05). Interestingly, the administration of CS/Res/Se-NPs-10 exhibited a potent anti-apoptotic effect with values comparable to those of the control group.

### Inhibitory effect of CS/Res/Se-NPs on key inflammatory cytokines

**Fig. 9:**
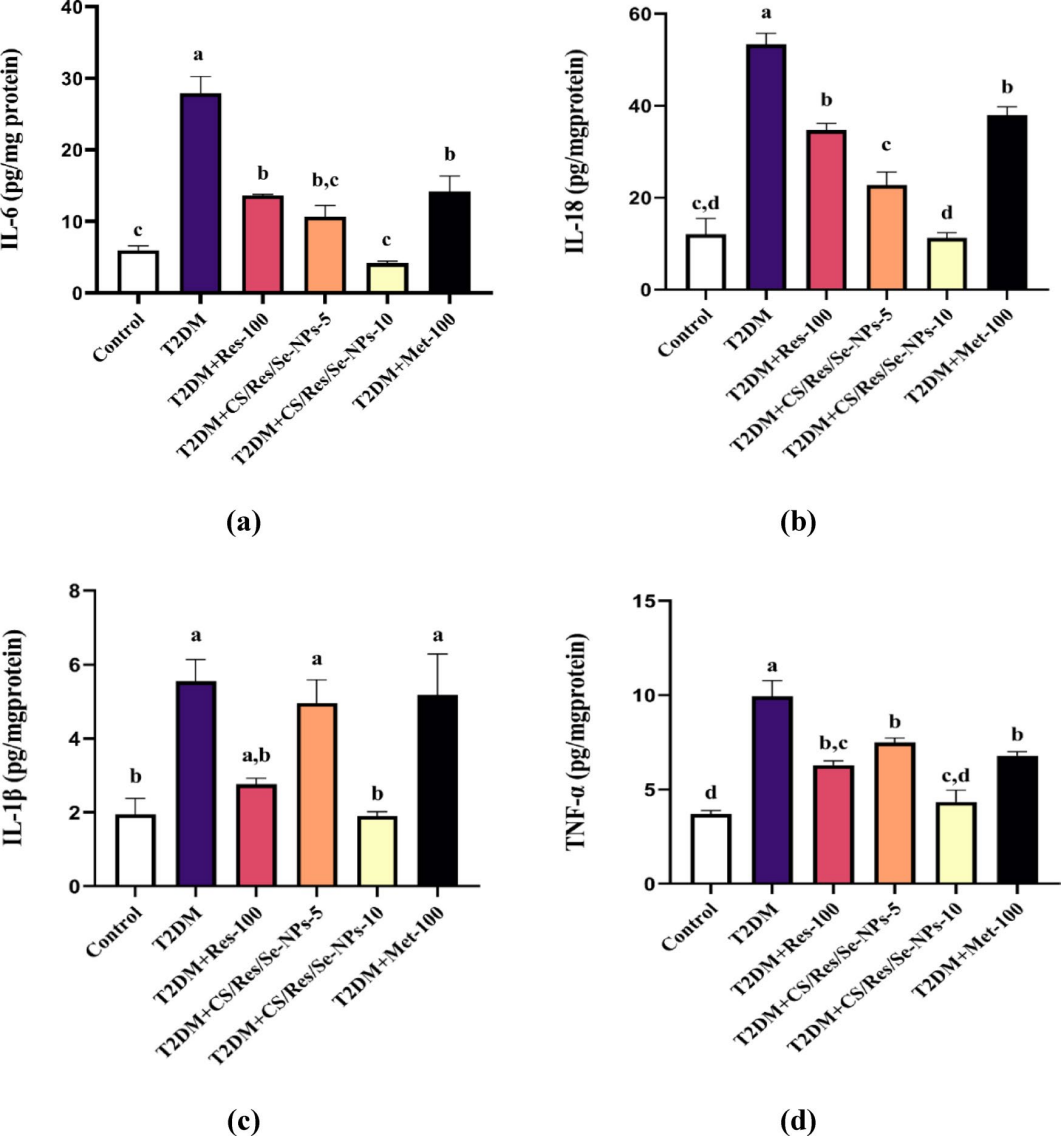

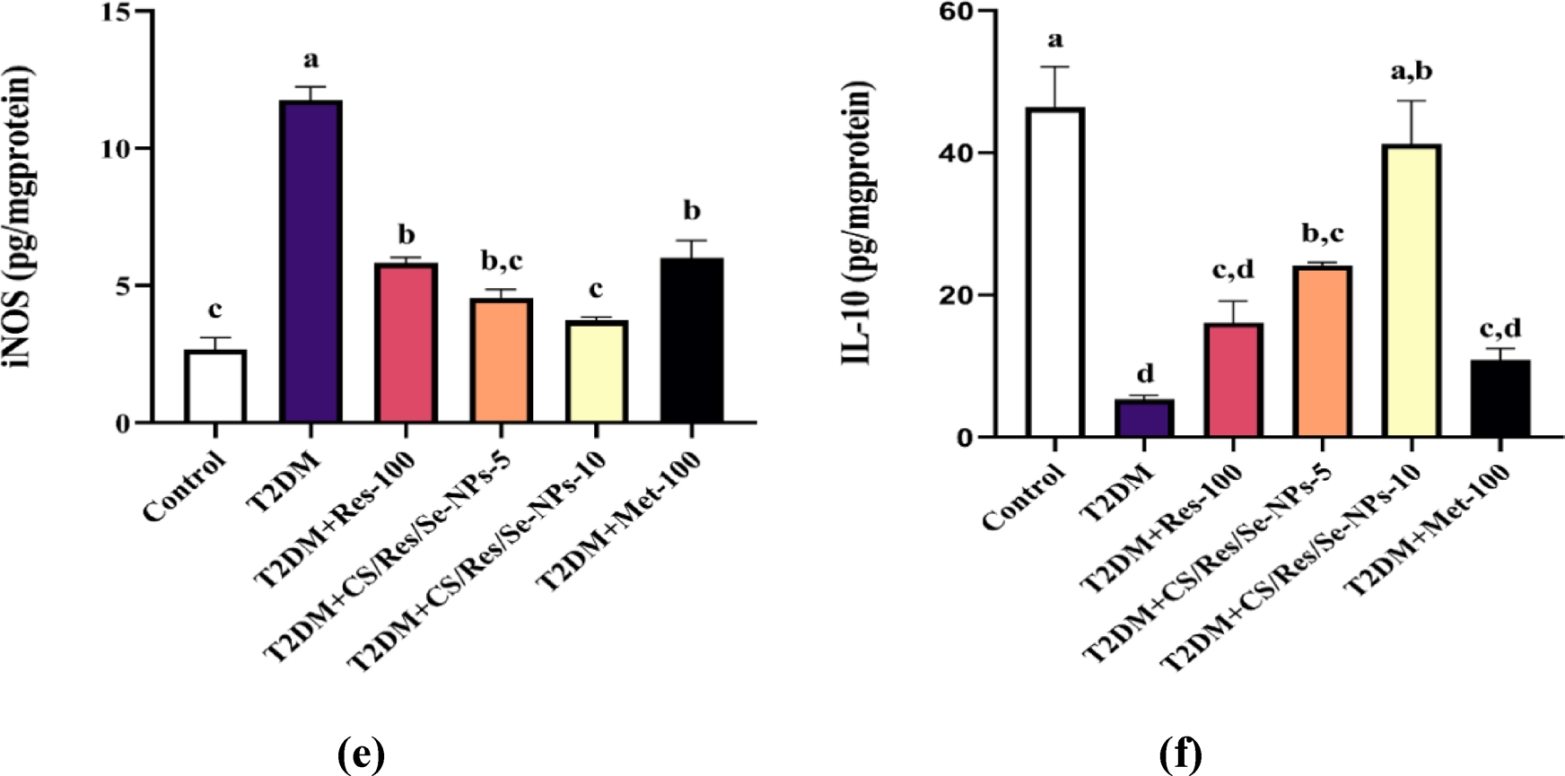
Changes in inflammatory biomarker levels in all studied groups. (**a**) IL-6 concentration, (**b**) IL-18 concentration, (**c**) IL-1β concentration, (**d**) TNF-α concentration, (**e**) iNOS concentration, and (**f**) IL-10 concentration. Data are means ± SEM (*n* = 6). Means with different letters in every bar indicate significant differences (Tukey, *p* < 0.05). The letter (a) is assigned to the highest data value, while the letter (d) is assigned to the smallest data value.

The current work demonstrated that the concentrations of hepatic pro-inflammatory cytokines, including IL-6, IL-18, IL-1β, TNF-α, and iNOS, were significantly elevated in mice given a 12-week HFD followed by STZ injection (5, 4, 3, 2.7, and 4.5-fold increase, respectively) compared to control mice (*p* < 0.05, Fig. 9a-e). Additionally, there was a significant reduction in the anti-inflammatory cytokine IL-10, which decreased by 9-fold compared to control mice (*p* < 0.05, Fig. 9f). These changes were strongly associated with the development of T2DM. In contrast, oral administration of CS/Res/Se-NPs-5 or CS/Res/Se-NPs-10, as well as standard Res-100 or Met-100, resulted in a significant reduction of the elevated concentrations of pro-inflammatory mediators, together with a marked increase in anti-inflammatory markers compared to the untreated diabetic mice. Notably, CS/Res/Se-NPs-10 exhibited a potent anti-inflammatory effect, with values comparable to those of the control group.

### Immunohistochemistry

**Fig. 10:**
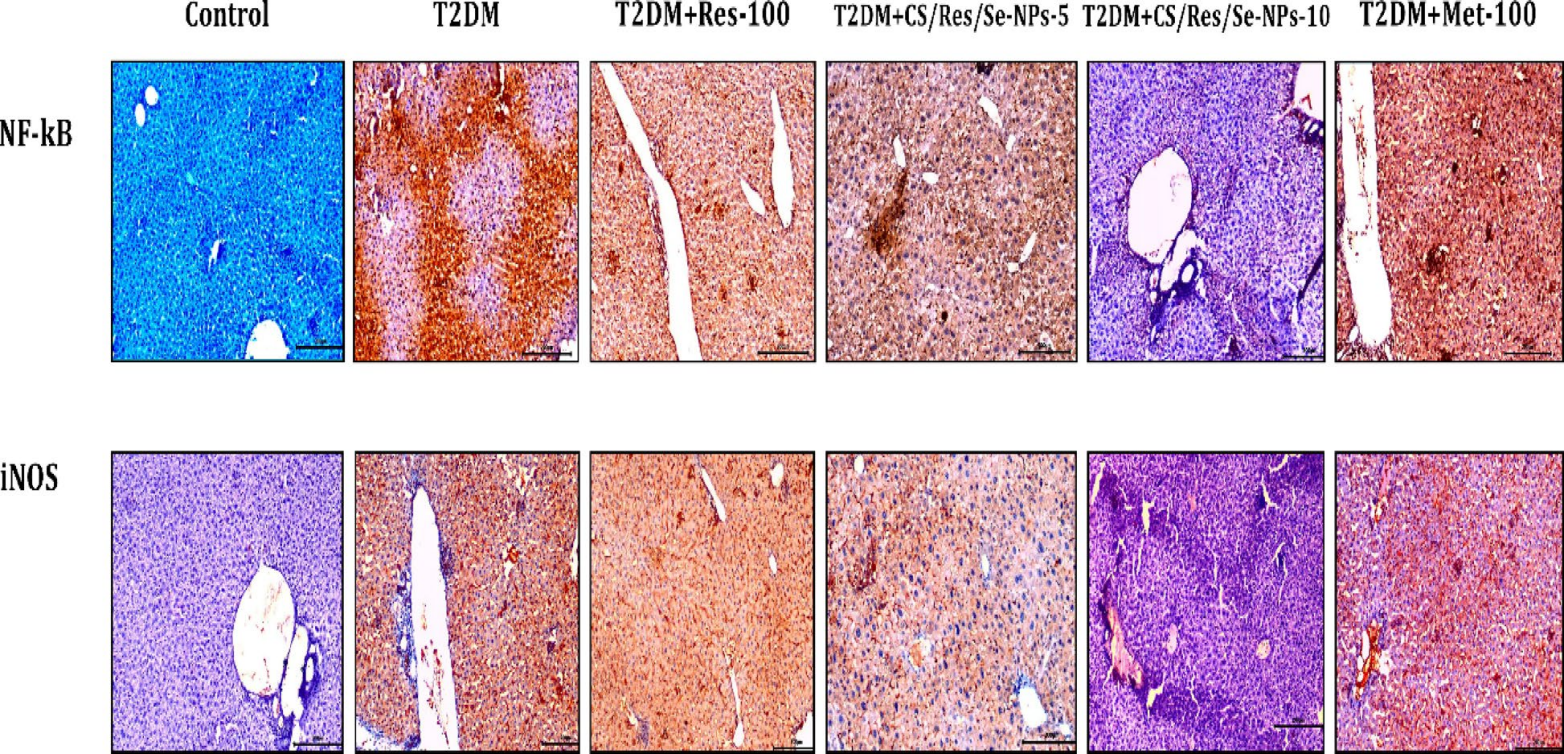
Photomicrograph of NF-κB and iNOS immune-stained liver sections, scale bar = 200 μm.

The effect of CS/Res/Se-NPs on the expression of NF-κB and iNOS in the liver tissues of normal and T2D mice was assessed using immunohistochemical staining. Liver sections from control mice showed no detectable immunoreactivity for either NF-κB or iNOS in the nuclei or cytoplasm (Fig. 10). In contrast, liver tissues from T2DM-induced diabetic mice exhibited strong immunoreactivity and widespread immunostaining for NF-κB and iNOS. In treated T2D groups, mice receiving Res-100 displayed faint to moderate widespread positivity for NF-κB and iNOS. Remarkably, CS/Res/Se-NPs-5 displayed faint to moderate diffuse positivity for NF-κB but strong and scattered positivity of iNOS in hepatic tissues. Notably, mice treated with CS/Res/Se-NPs-10 showed very faint and limited NF-κB staining, along with minimal iNOS expression. On the other hand, Met-100-treated mice exhibited strong to moderate widespread immunoreactivity for both NF-κB and iNOS in hepatic tissue. These findings indicate that CS/Res/Se-NPs, particularly at the higher dose (CS/Res/Se-NPs-10), effectively reduced the expression of NF-κB and iNOS in the liver tissues of T2D mice.

### Histological examination of liver

**Fig. 11:**
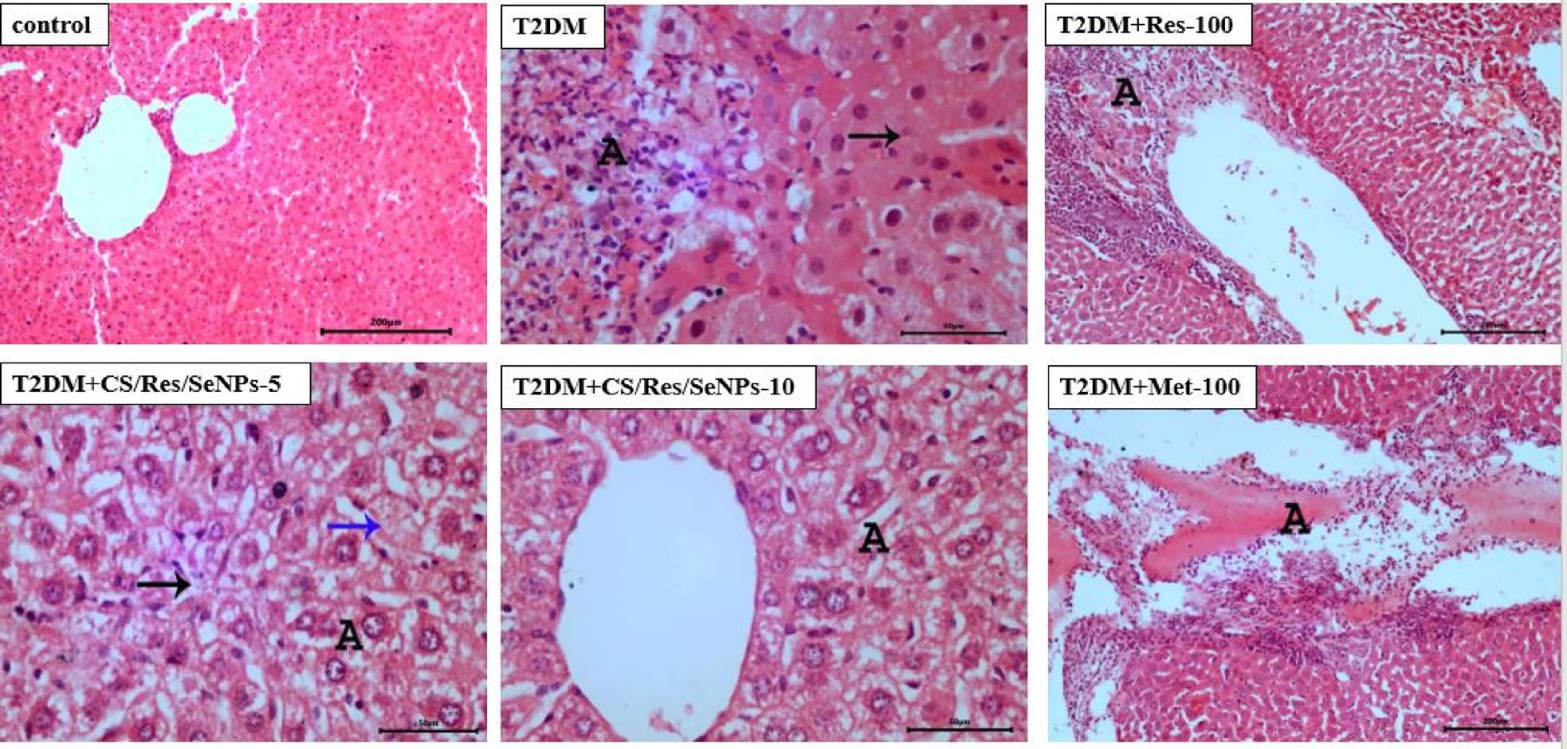
Photomicrographs of hepatic sections in all studied groups (H&E- Mag×100). Liver sections of control mice with normal histo-architecture, on contrary, liver sections of T2DM-induced mice with mononuclear inflammatory cells infiltration (A) and pyknotic nuclei. T2DM + Met-100 liver sections with accumulation of mononuclear inflammatory cells (A). Likewise, T2DM + Res-100 with accumulation of mononuclear inflammatory cells (A). Vacuolar degeneration was obvious upon examination of liver sections of both T2DM + CS/Res/Se-NPs-5 and T2DM + CS/Res/Se-NPs-10 treated groups, but T2DM + CS/Res/Se-NPs-5 with minimal focal hepatocytes degeneration (A) and infiltration of mononuclear inflammatory cells (black arrow) besides presence of some hepatocytes with karyolysis (blue arrow).

As shown in Fig. 11, liver sections of control mice via light microscope showed that both of portal areas and hepatocytes appeared with normal histo-architecture. In contrast, T2DM-induced mice liver displayed destruction of the normal architecture of the hepatocytes, as well as showed several areas of hepatocyte necrosis with mononuclear inflammatory cells infiltration and presence of some hepatocytes with faint pyknotic nuclei. However, a significant intravascular and perivascular buildup of mononuclear inflammatory cells was observed in the liver sections of T2DM + Met-100 treated group. Similarly, the T2DM + Res-100-treated group showed peri-vascular accumulation of mononuclear inflammatory cells. Furthermore, both the T2DM + CS/Res/Se-NPs-5 and T2DM + CS/Res/Se-NPs-10 liver sections displayed vacuolar degeneration, but T2DM + CS/Res/Se-NPs-5 treated group also had some hepatocytes with karyolysis, with minimal focal hepatocyte degeneration and infiltration of mononuclear inflammatory cells.

The results of histopathological evaluation of the hepatic lesions of different groups using a simple semi-quantitative scoring system are shown in Table 6. Briefly, five random fields from each animal liver histopathological sections were examined (×100), the grade of the detected lesion severity was assessed depending on the percentage of affected area/entire section and recorded as follow: (-): absence of lesion, (+): for mild degree of lesions (5–25%), (++): for moderate lesions degree (26–50%) and (+++): for severe degree of lesions (≥ 50%).

**Table 6:** Semi-quantitative grading system for hepatic histopathological alterations.

Scored liver lesions	Incidence^1^ and Severity^2^ of histopathological lesions																			
	T2DM-induced mice				T2DM + Res-100				T2DM + CS/Res/Se-NPs-5				T2DM + CS/Res/Se-NPs-5				T2DM + Met-100			
	Absent (-)	Mild (+)	Moderate (++)	Severe (+++)	Absent (-)	Mild (+)	Moderate (++)	Severe (+++)	Absent (-)	Mild (+)	Moderate (++)	Severe (+++)	Absent (-)	Mild (+)	Moderate (++)	Severe (+++)	Absent (-)	Mild (+)	Moderate (++)	Severe (+++)
1-Hepatocytes vacuolar degeneration	1	1	1	3	2	1	1	2	2	2	1	1	2	3	2	1	1	2	1	2
2-Perivascular Inflammatory cells infiltration	0	3	3	0	2	2	1	1	1	3	2	0	4	2	0	0	0	1	1	4
3-Hepatocellular necrosis	0	1	2	3	1	2	1	2	2	3	1	0	4	1	1	0	2	0	1	3
4-Hepatocytes with pyknotic nuclei	1	3	1	1	3	3	0	0	3	3	0	0	5	0	1	0	2	1	3	0
5- Hepatocytes with karyolysis	2	1	3	0	4	2	0	0	5	1	0	0	5	1	0	0	4	2	0	0

^1^Number of mice with lesions per total examined (6 mice).

^2^Severity of lesions was graded by estimating the percentage area affected in the entire section.

### Molecular docking study

**Fig. 12:**
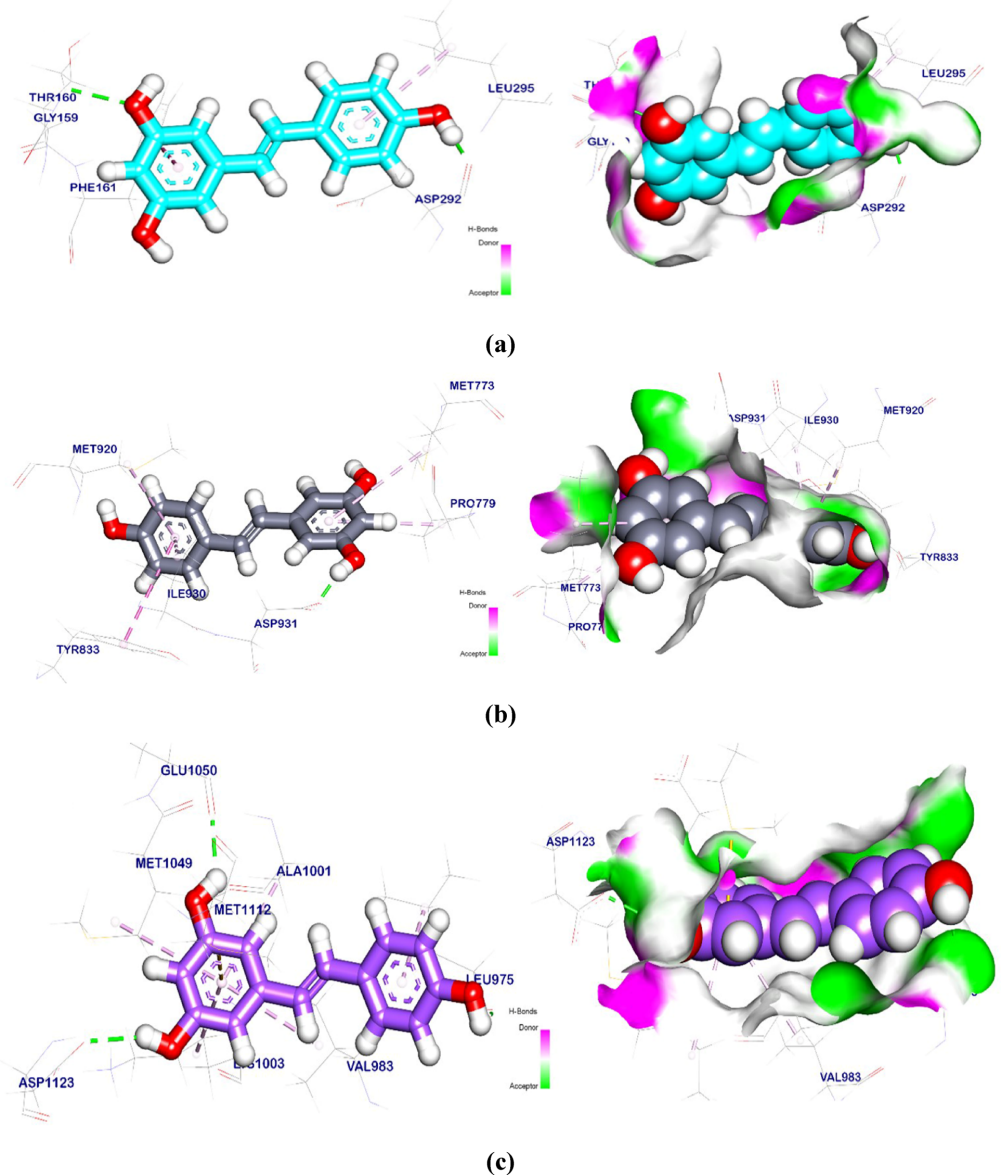

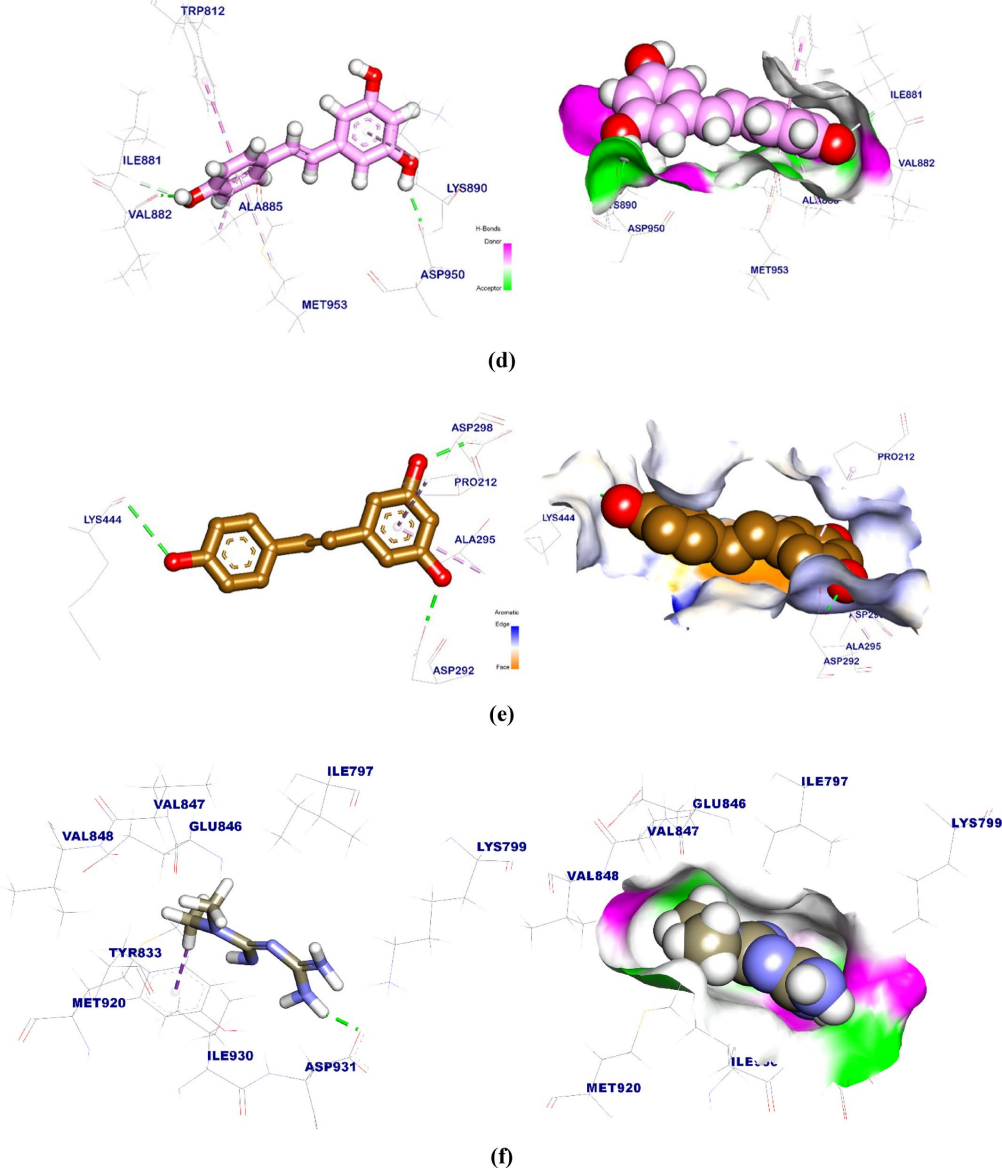
3D and surface mapping of Res against protein target site. **(a)** AKT, **(b)** PI3K-β, **(c)** IGF-1, **(d)** mTOR, and **(e)** SIRT-1 and **(f)** Met against PI3K-β.

The binding mode of Res exhibited an energy binding of −5.58 kcal/mol against AKT target site. Res formed one Pi-Alkyl and one Pi-Pi interactions with Phe161 and Leu295. Moreover, Asp292 and Thr160 interacted with Res by two hydrogen bonds with bond lengths of 1.91 and 2.53 Å (Fig. 12a). The binding mode of Res exhibited an energy binding of −5.98 kcal/mol against PI3K target site. Res formed four Pi-Alkyl and one Pi-Pi interactions with Tyr833, Met920, Ile930, Pro779 and Met773, additionally interacting with Asp931 by two hydrogen bonds with a distance of 2.14, 2.03 and 1.97 Å (Fig. 12b). The binding mode of Res exhibited an affinity binding energy of −5.67 kcal/mol against the IGF-1 target site. Res binds with five Pi-Alkyl and one Pi-sulfur interactions with Lys1003, Ala1001, Met1049, Val983, and Met1112, additionally, it formed three hydrogen bonds with Asp1123, Glu1050, and Leu975 with bond lengths of 2.37, 2.16, and 2.81 Å (Fig. 12c). The binding mode of Res exhibited an energy binding of −5.60 kcal/mol against mTOR target site. It formed three Pi-Alkyl and one Pi-Pi interactions with Lys890, Met953, and Ala885; moreover interacted with Val822 and Asp950 by two hydrogen bonds with bond lengths of 2.06 and 2.22 Å (Fig. 12d). The binding mode of Res exhibited an energy binding of −7.90 kcal/mol against SIRT-1 target site. Res formed two Pi-Alkyl interactions with Pro212 and Ala295, on the other hand, Res interacted with Asp292, Asp298, and Lys444 by three hydrogen bonds with bond lengths of 2.27, 2.26, and 3.31 Å (Fig. 12e). Furthermore, the proposed binding mode of Met against PI3K-β exhibited an affinity score equal to −4.84 kcal/mol. Met formed one pi-sigma interaction and one hydrogen bond with Tyr833 and Asp 931, with a bond length of 2.02 Å (Fig. 12f). The molecular docking was done, twenty poses were generated, then the best orientations were captured, and affinity scores and RMSD values were collected in Table 7.

**Table 7:** Shows (DG, RMSD, interactions) kcal/mol of res and met.

Targets	Tested compounds	RMSD value (Å)	Docking (Affinity) score (kcal/mol)	Interactions	
				H.B Pi -interaction	
AKT	**Res**	0.47	−5.85	2	2
PI3K-β		0.78	−5.98	2	5
IGF-1		0.94	−5.67	3	6
mTOR		0.77	−5.60	2	4
SIRT-1		0.75	−7.90	3	2
PI3K-β	**Met**	1.23	−4.84	1	0

### Molecular dynamics (MD) simulation studies

#### Protein and ligand RMSD analysis

**Fig. 13:**
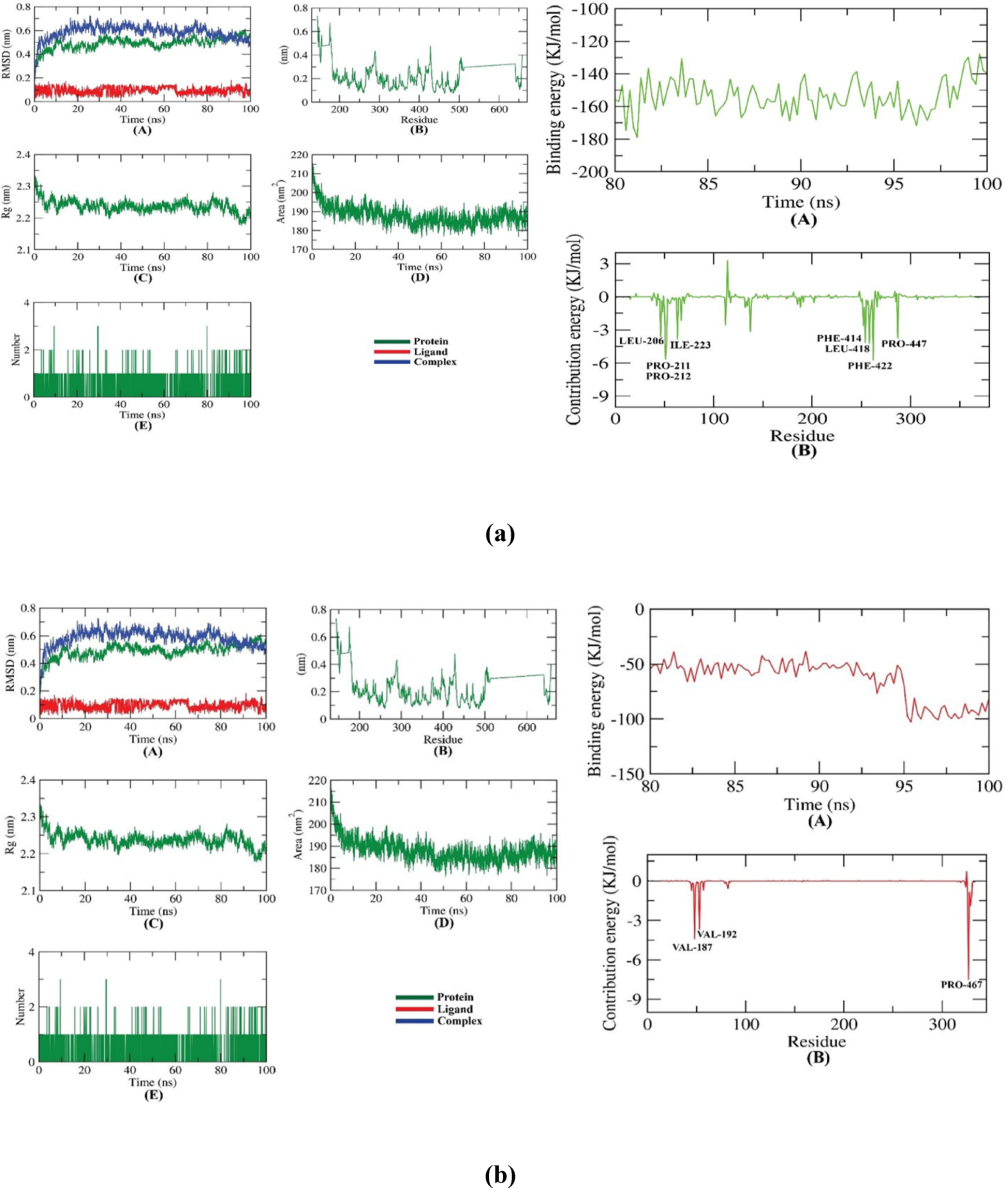
(**a**) The RMSD of the Res-SIRT complex and (**b**) The RMSD of the Res-AKT complex.

For MD simulations, Res was complexed with SIRT as shown in Fig. 13a. The dynamic motion and conformational changes of the backbone atoms of the protein-ligand complex were calculated using RMSD to ascertain their stability in the apo and ligand-bound states. The protein, ligand, and complex are observed to have a lower RMSD with no discernible variations during the simulation, indicating greater stability. The flexibility of each residue was determined using RMSF to have a better knowledge of the region of proteins that fluctuates during the simulation. Ligand binding does not cause any protein residues to become flexible. The radius of gyration demonstrated the compactness of the complex (Rg). It was found that the complex’s Rg was lower than its original period, indicating that it is becoming more stable and compact. The solvent-accessible surface area was used to evaluate the interaction between protein-ligand complexes and solvents during the simulation phase (SASA). SASA of the complex was established to assess the degree of conformational alterations that occurred throughout the interaction. It’s interesting to note that the protein showed a decrease in surface area with a relatively lower SASA value compared to its early stage. For a protein and ligand to form hydrogen bonds, the complex must be structurally stable. The majority of protein conformations were found to create up to two hydrogen bonds with the ligand. Using MM/PBSA, we calculated the binding free energy of the protein-ligand complex’s last 20 ns of MD production run with an interval of 100 ps from MD trajectories. MmPbSaStat.py calculated the average free binding energy and standard deviation/error from g MMPBSA output files. Protein-ligand binding free energy was − 153 kJ/mol. We also calculated each protein residue’s binding free energy contribution to the ligand interaction. Decomposing the system’s total binding free energy into per-residue contribution energy and calculating each residue’s contribution. The binding energy remained consistently negative throughout the simulation, with an average value of approximately − 153 kJ/mol. This stability indicates a strong and energetically favorable interaction between the ligand and the receptor, reflecting the stability of the complex during the MD simulation. This revealed the “crucial” residues that aid protein-molecule binding. LEU-206, PRO-211, PRO-212, ILE223, PHE-414, LEU-418, PHE-422, and PRO-447 residues of the protein contributed more than − 5 kJ/mol binding energy and are hotspot residues in ligand binding.

In addition, Res was complexed with AKT as shown in Fig. 13b. RMSD calculations were utilized to analyze the protein-ligand complex’s apo and ligand-bonded stability by determining the backbone atoms’ dynamic atom motions and conformational changes. The protein, ligand, and complex show decreasing RMSD with no significant fluctuations during the simulation, demonstrating stability. Root-mean-square fluctuation (RMSF) was determined for each residue to better understand protein regions fluctuating throughout the simulation. It makes sense that ligand binding renders the protein flexible between 350 and 400 residues. The radius of gyration showed the complex’s compactness (Rg). A compact system has reduced fluctuation during simulation. The complex had a lower Rg than in the prior period. The simulation’s solvent-accessible surface area (SASA) measured solvent-protein-ligand complex interactions. The complex’s SASA was measured to determine the interaction’s conformational alterations. Intriguingly, the protein’s surface area and SASA score decreased. Hydrogen bonding is necessary for protein-ligand order. Most protein conformations form two hydrogen bonds with the ligand. We calculated the binding free energy of the protein-ligand complex during the final 20 ns of the MD production run with an interval of 100 ps using MM/PBSA and MD trajectory data. From the g mmpbsa output files, MmPbStat.py calculated the average free binding energy and standard deviation/error. The free energy of binding a protein ligand was − 64 kJ/mol. Additionally, we calculated the binding free energy contribution of every protein residue to the ligand interaction. The binding energy fluctuated between − 50 and − 100 kJ/mol; however, a marked decrease occurred around 92 ns, after which the energy stabilized near − 120 kJ/mol. This shift suggests a structural rearrangement resulting in a more favorable binding conformation. Dividing the total binding free energy of the system into the energy contributed by each residue. The “crucial” residues that facilitate protein-molecule binding were thus identified. The protein’s VAL-187, VAL-192, and PRO-467 residues are hotspot residues for ligand binding because they contributed more than − 3 kJ/mol of binding energy.

## Discussion

Type 2 diabetes mellitus is a complicated endocrine condition characterized by a cascade of biochemical and cellular processes, such as liver injury, pancreatic β-cell damage, oxidative stress, as well as disturbances in protein and gene expression. Given the difficulties of treating all of the aforementioned events with a single medicine, it is appropriate to seek out a powerful nutritional therapy capable of properly managing the majority of the detrimental events associated with this metabolic condition. Phytochemical-rich herbal therapies are widely utilized in many underdeveloped countries, with approximately four billion individuals relying on plant-based medicine to manage metabolic disorders, including diabetes mellitus. So, it is crucial to understand and record the function of plant-based phyto-compounds in managing diabetes and controlling blood glucose levels. Res has been scientifically shown to offer several health benefits. However, it has low bioavailability in humans due to its extensive metabolism and rapid elimination^30^. Once Res is released and absorbed into the bloodstream, it is recognizable as a degraded and unmodified compound^31^. In addition to the anti-diabetic properties of traditional Res, the utilization of SeNPs has been previously documented to exhibit negligible and undetectable cytotoxicity or liver toxicity, in addition to their notable biological activity and high bioavailability^14^. Hence, the current investigation aimed to identify a suitable pharmaceutical product utilizing nanoformulations of CS/Res/SeNPs to alleviate diabetic complications and adverse alterations in HFD/STZ-induced T2DM in mice.

Researchers are trying to create novel Res-based nanotechnologies that are more efficient and bioavailable to address these challenges. In our study, the CS/Res/SeNPs preparation and characterization study was piloted as the first step. TEM analysis of the nanoparticle architecture revealed that the CS/Res/SeNPs had a larger particle size compared to free CS, confirming that Res was effectively loaded into the CS nanoparticles, which was in line with previous research^32^. On the other hand, several distinctive peaks of Res were greatly diminished, and the absorption intensity of these specific bands had significantly disappeared. This implies that Res was enclosed by a hydrophobic core of the nanoparticle, which prevented Res’s various bonds from bending and stretching. Additionally, the hydroxyl group peaks were moved to 3416.84 cm^−1^, which may be because of the hydrogen bonds that were formed between the phenolic groups in Res and the amine groups of CS^33^. Moreover, free CS had a zeta potential of + 20.6 mV, most likely as a result of its positively charged -NH3^+^ functional groups. However, due to the numerous CS amino groups, CS increased the zeta potential of SeNPs in CS/SeNPs up to + 26.1 mV. In contrast, the zeta potential values for CS/Res/SeNPs were moved from + 20 to + 10.9 mV. The O-H groups in the structure of Res may be the cause of this displacement^34^.

To quickly hasten the onset of T2DM as well as induce gradual and mild destruction of β-cells, HFD has been used in conjunction with multiple low doses of STZ^35^. Our results confirmed that feeding HFD for 12 weeks contributed to obesity. This has been associated with weight gain, high body mass index, and a likelihood of IR, as well as hyperlipidemia, together with hypercholesterolemia. Furthermore, multiple low doses of STZ injections are known to stimulate gradual β-cell destruction accompanied by hyperglycemia, IR, and dyslipidemia. The current results revealed that the HFD/STZ-induced T2DM group showed symptoms of excessive diuresis, and excessive or abnormal thirst, along with a notable reduction in body weight, which aligns with our previous research^36,37^. The drop in body weight could be simply because of a lack of insulin, muscle breakdown, structural protein deterioration, or due to a reduction of the total protein content in muscle via proteolysis.

Treating mice with CS/Res/Se-NPs greatly improved glycemic control by boosting insulin sensitivity as well as lowering FBG and fasting insulin concentrations, along with increased synthesis of structural proteins^38,39^. Previously published investigations on isolated rat adipocytes revealed that sodium selenate triggers the activation of serine/threonine kinases, particularly the p70 S6 kinase, thereby promoting glucose absorption via glucose transporters’ translocation to the plasma membrane^40,41^. In addition, the ability of Res to interact with specific targets in the insulin signaling pathway was evaluated, confirming a potent binding affinity. The results from the docking study confirm the hypoglycemic actions of Res in T2D-induced mice via induction of the activities of PI3K/AKT/mTOR pathways^42,43^. The anti-diabetic effect of CS/Res/Se-NPs could be because of its in vivo stimulation of SIRT-1 expression, which is a NAD^+^-dependent deacetylase that works after caloric restriction and has a positive impact on glucose management^44^. As well, Se has the potential to induce insulin-mimetic effects by activating AKT as well as various kinases that are involved in the insulin signaling cascade^45^. Additional mechanisms observed in vitro for Se, including the enhancement of renal glucose excretion and the inhibition of intestinal glucose transport, may substantiate the hypoglycemic effect of Se^46^.

Diabetes-related hyperglycemia elevates the likelihood of liver injury, which is one of the most frequently occurring issues that can happen with diabetes^47^. Liver aminotransferases serve as highly sensitive biomarkers of liver damage. Our data showed that HFD followed by multiple low doses of STZ contributed to a considerable increase in liver enzymes ALT, AST, as well as ALP, with a substantial reduction in the concentrations of albumin and total protein, relative to control mice^48^. The observed hepatic injury in HFD/STZ-induced mice was reversed towards normal values by treatment with CS/Res/SeNPs, confirming the hepatoprotective effect of Res^49^. Res’s hepatoprotective effects may be attained by restoring the liver cell membrane and accelerating parenchyma regeneration. This will guard the liver against its membrane fragility and prevent leakage of liver enzymes into the serum of T2DM-induced mice^50^. The notable hepatoprotective actions of SeNPs contribute effectively to their ability to scavenge free radicals, preserve liver tissue integrity, and improve liver functions^14,51^.

T2DM is also associated with severe kidney injury, probably due to acute mitochondrial dysfunction and hyperglycemia^52^. HFD causes alterations in lipid metabolism in the kidney by creating an imbalance between lipid synthesis and its breakdown, together with systemic metabolic disturbances, and their corresponding renal fat buildup and kidney damage^53^. Beyond this, STZ triggers direct toxicity to the kidneys, and they concluded that the treatment action ought to be delayed at least 3 weeks till renal recovery from STZ’s acute nephrotoxic impacts^54^. STZ-induction enhanced instability in renal histomorphological architecture and significantly elevated creatinine and urea serum concentrations, which is consistent with previous studies^55^. Considering the above findings, our results demonstrated that after oral administration of CS/Res/SeNPs, a remarkable decrease in both creatinine and urea serum levels was observed relative to the untreated diabetic mice^56^. The significant decline in creatinine and urea levels in our current study may be due to the action of Res and SeNPs on increasing antioxidant enzyme activity and alleviating ROS-induced renal oxidative damage and inflammation^57^.

As previously confirmed, DM-associated lipid metabolic disturbances are often defined by increased serum lipid concentrations^58,59^. HFD consumption has been associated with dyslipidemia and disturbances in the expression of genes involved in lipid metabolism within the liver^49^. Current findings demonstrated that the HFD/STZ-induced mice had remarkable dyslipidemia, as confirmed by a substantial rise in the serum lipid profile parameters. This dyslipidemia might have been triggered by HFD intake, as confirmed by a considerable increase in the lipid profile parameters, which may be additionally metabolized, yielding glycerol or acetyl-CoA, which both assist in the hepatic fat accumulation and TG synthesis^60^. Our findings showed that CS/Res/SeNPs supplementation exerted significant effects on lowering blood LDL, TC, and TG and increasing HDL concentrations^61,62^. Res’s curative properties may be due to its capacity to reduce 3-hydroxy-3-methylglutaryl-coenzyme A reductase (HMG-CoA reductase) mRNA expression, assist in the transition of lipid metabolism to oxidation, induce remodeling of myocellular lipid stores, reduce lipid accumulation, by promoting lipolysis in adipocytes^63,64^.

Oxidative stress signaling pathways are the first stage in the development of DM. The administration of HFD and STZ injection could contribute to oxidative damage within β-cells of the pancreas. ROS-induced oxidation of lipid components produces MDA, a byproduct of lipid peroxidation and a key component of TBARS^65^. Remarkably, Res’s anti-oxidant properties are one of the primary strategies underlying its anti-diabetic benefits. Our data demonstrated that, in comparison to conventional Res and Met, the course of treatment with CS/Res/Se-NPs-10 showed a more therapeutic effect via boosting the hepatic enzymatic and non-enzymatic antioxidants with a substantial decline in oxidative stress indicator levels among T2DM-induced mice^66^. Earlier studies have shown that a diet containing either SeNPs caused an increase in SOD, GPx, and CAT activities, which may be due to its enhanced absorption efficiency primarily via the large surface area and small size of Se nanoparticles^14,67,68^.

Diabetes may induce apoptosis through increased oxidative stress and elevated inflammatory cytokines. Therefore, it would be rational to expect an increase in hepatic expression of apoptotic proteins in HFD/STZ-induced mice^69^. Res has been shown in some studies to have positive impacts on apoptotic biomarkers in STZ-induced diabetic mice liver^70^. Notably, CS/Res/SeNPs supplementation, relative to other therapy options, demonstrated a higher anti-apoptotic potency and exhibited considerable suppression of hepatic expression of Bax, caspase-3, and caspase-8, whereas a substantial elevation of Bcl-2 expression^71^. Res may have an anti-apoptotic impact because it promotes mitochondrial antioxidant enzyme activity like SOD2, which lessens oxidative damage brought on by apoptosis^72^. Res may also act by stimulating SIRT-1 proteins, which in turn enhance mitochondrial function and inhibit apoptosis^73^. In addition to Res, SeNPs may mitigate the increase in IL-6 and TNF-α gene expressions, subsequently decreasing hepatic apoptosis and inflammation^14,74,75^.

Feeding HFD to mice not only increased the expression of genes related to the intrinsic and extrinsic apoptotic cascade, but it also boosted the expression of inflammatory biomarkers in the liver relative to control mice. Chronic inflammation is an essential contributor to diabetes onset and its associated complications. Several pro-inflammatory cytokines are linked to reduced insulin production and contribute to the development of T2DM. It is obvious from our results that HFD/STZ increased hepatic pro-inflammatory cytokines IL-1β, TNF-α, IL-6, and IL-18, as well as iNOS concentrations^14^. The oral administration of CS/Res/SeNPs could stimulate the anti-inflammatory mediator IL-10 and reduce pro-inflammatory protein levels in diabetic animals, which is in agreement with other studies^76–78^. Res’s suppressing and targeting of the NOD-like receptor protein 3 (NLRP3) inflammasome emphasize its anti-inflammatory properties^79^. In addition, a previous study indicated that SeNPs inhibited levels of mitogen-activated protein kinase (MAPK), NF-κB, and TNF-α in rats^80^. Our immunohistochemistry analysis further supported these findings, revealing extensive positivity for NF-κB and iNOS in the liver sections of T2DM-induced mice. In contrast, the treated groups displayed only limited and faint staining for both markers, especially CS/Res/Se-NPs-10 treated group.

Finally, our histopathological results were consistent with the biochemical and molecular analysis. In HFD/STZ-induced animals, the microarchitecture of the hepatic tissues was significantly injured. The histological examination revealed that CS/Res/Se-NPs had an ameliorating impact on hepatic tissues, suggesting that cell architecture had been restored. Remarkably, our data showed that mice treated with CS/Res/Se-NPs-10 had a better outcome; these animals showed mild repair of the hepatocytes’ structure, in addition to a significant improvement in liver profile marker levels, demonstrating the hepatoprotective and therapeutic role of the prepared NPs. The strong antioxidant and anti-inflammatory properties of the prepared NPs may mediate these positive outcomes^81^. These findings suggest that CS/Res/Se-NPs could be used to alleviate or treat T2DM-related complications.

## Conclusion

In summary, the current data revealed that CS/Res/Se-NPs-10 exhibited the most potent antioxidant, anti-diabetic, and anti-inflammatory activities along with remarkable hepatoprotective effects over conventional Res/Met (Table 8). In this context, it demonstrated a more favorable impact on T2DM development by enhancing hepatic antioxidant enzyme activity and significantly reducing oxidative stress biomarkers. This suggests that CS/Res/SeNPs could offer a novel strategy for improving T2DM management by normalizing serum biochemical parameters, treating hyperglycemia, improving insulin sensitivity, and attenuating oxidative stress, inflammation, and apoptosis in liver tissues. Furthermore, in silico study demonstrated that hydrogen bond formation was pivotal in the interaction between Res and insulin signaling target proteins such as AKT, PI3K-β, IGF-1, mTOR, and SIRT-1. Moreover, our immunohistochemistry analysis further confirmed these observations, demonstrating that treatment with CS/Res/SeNPs exhibited markedly reduced NF-κB and iNOS expression in liver sections. Our data suggests that CS/Res/SeNPs could serve as a promising and effective delivery system to enhance the therapeutic impact of Res as an oral treatment of T2DM and its complications.

**Table 8:** Comparative table summarizing the key improvements of CS/Res/Se-NPs-10 over conventional Res/Met, emphasizing improved biochemical outcomes.

Property	Conventional Res/Met	CS/Res/SeNPs-10	Improvement
Antioxidant activity	Moderate	Significantly higher	✓↑ ROS scavenging and oxidative stress reduction
Anti-diabetic action	Less effective in glycemic control	Greater reduction in glucose levels, ↑ Glucose homeostasis, and insulin sensitivity	✓ Stronger therapeutic outcome
Anti-inflammatory effect	Moderate reduction	Significant downregulation	↓ Pro-inflammatory cytokines and ↑ anti-inflammatory cytokines
Liver and renal profile	Slight improvement	Improved protection and normalization	↑ Hepatorenal protection
Toxicity	Higher at therapeutic doses	Lower at therapeutic dose	✓ Better safety profile
Apoptotic markers	Moderately downregulated	Significantly normalized	↑ Strong anti-apoptotic effect
Histopathological features	Partial tissue protection	Near-normal architecture, reduced necrosis, and inflammation	↑ Superior tissue protection and repair

## Electronic supplementary material

Below is the link to the electronic supplementary material.

Supplementary Material 1

## Data Availability

The authors declare that the data supporting the findings of this study are available within the manuscript.
