# Supplementary material for: Biochemical and molecular evaluation of resveratrol and selenium nanoparticles in managing type 2 diabetes and its complications
Source: Sci Rep. 2025 Jul 15;15:25565. doi: 10.1038/s41598-025-11156-x (PMC12264291; doi:10.1038/s41598-025-11156-x)
Supplement: Supplementary file 1 — Supplementary Material 1 [file 41598_2025_11156_MOESM1_ESM.docx]

**Table S1. Body weight of animals in the different groups.**

| **Groups** | **Weeks** | | | | | | | | | | | | | | | | | | | | | |
| --- | --- | --- | --- | --- | --- | --- | --- | --- | --- | --- | --- | --- | --- | --- | --- | --- | --- | --- | --- | --- | --- | --- |
|  | **1** | **2** | **3** | **4** | **5** | **6** | **7** | **8** | **9** | **10** | **11** | **12** | **13** | **14** | **15** | **16** | **17** | **18** | **19** | **20** | **21** |  |
| **Control** | 17.97±0.32^a,b^ | 19.43±0.23^b^ | 20.57±0.38^b^ | 22.23±0.19^c^ | 23.43±0.22^c^ | 24.13±0.15^c^ | 25.17±0.12^c^ | 26.43±0.20^b,c^ | 27.3±0.17^c^ | 28.2±0.12^c^ | 29.13±0.13^c^ | 29.9±0.06^c^ | 30.43±0.23^c^ | 31.5±0.20^c^ | 32.3±0.15^c^ | 32.6±0.31^b^ | 33.03±0.55^a,b^ | 31.57±0.43^b^ | 32.5±0.29^a^ | 32.07±0.18^a^ | 32.9±0.21^a^ |  |
| **T2DM** | 14.43±0.43^c^ | 15.4±0.17^d^ | 15.6±0.35^d^ | 16.6±0.21^f^ | 18.47±0.26^e^ | 20.57±0.72^d^ | 23.33±0.55^d^ | 28.1±0.59^b^ | 30.03±0.58^b^ | 33.37±0.45^b^ | 35.43±0.38^a,b^ | 37.83±0.46^a^ | 40.8±0.99^a^ | 38.93±0.35^a^ | 37.47±0.37^a^ | 36.47±0.29^a^ | 34.93±0.22^a^ | 33.77±0.38^a^ | 33.4±0.31^a^ | 31.63±0.63^a^ | 31±0.58^a^ |  |
| **T2DM+Res-100** | 15.8±0.42^b,c^ | 17.83±0.22^c^ | 18.63±0.19^c^ | 19.27±0.15^d^ | 20.3±0.21^d^ | 21.6±0.35^d^ | 23.1±0.26^d^ | 24.6±0.21^c,d^ | 25.97±0.18^c,d^ | 27.8±0.26^c^ | 29.1±0.31^c^ | 30.6±0.35^b,c^ | 32.63±0.32^b,c^ | 32.5±0.32^c^ | 31.17±0.17^c,d^ | 29.83±0.09^c^ | 29.07±0.18^c,d^ | 28.33±0.28^c^ | 27.17±0.33^b^ | 25.47±0.27^c^ | 24.13±0.32^b,c^ |  |
| **T2DM+CS/Res/Se-NPs-5** | 19.1±0.59^a^ | 20.9±0.10^a^ | 21.87±0.47^b^ | 23.73±0.18^b^ | 25.1±0.15^b^ | 26.87±0.35^b^ | 28.93±0.46^b^ | 30.4±0.31^a^ | 32.73±0.18^a^ | 33.53±0.26^b^ | 35.27±0.27^b^ | 37.17±0.44^a^ | 39.1±0.46^a^ | 36.77±0.23^b^ | 34.2±0.42^b^ | 32.13±0.41^b^ | 31.07±0.56^b,c^ | 29.97±0.58^b,c^ | 27.87±0.47^b^ | 27.47±0.33^b^ | 25.6±0.31^b^ |  |
| **T2DM+CS/Res/Se-NPs-10** | 17.6±0.42^a,b^ | 20.5±0.29^a^ | 23.53±0.32^a^ | 26.07±0.12^a^ | 28.13±0.59^a^ | 29.2±0.15^a^ | 30.8±0.47^a^ | 32.43±0.69^a^ | 33.9±0.10^a^ | 35.4±0.23^a^ | 36.87±0.41^a^ | 38±0.23^a^ | 39.4±0.36^a^ | 37.7±0.30^a,b^ | 35.17±0.66^b^ | 32.83±0.60^b^ | 30.1±0.67^c^ | 28.77±0.39^c^ | 27.67±0.33^b^ | 26.17±0.73^b^ | 22.83±0.27^c,d^ |  |
| **T2DM+Met-100** | 14±0.58^c^ | 15.57±0.22^d^ | 16.53±0.19^d^ | 17.53±0.23^e^ | 18.67±0.44^d,e^ | 20.3±0.35^d^ | 21.8±0.15^d^ | 23.73±0.41^d^ | 25.9±0.29^d^ | 28.03±0.26^c^ | 29.37±0.37^c^ | 31.9±0.38^b^ | 33.83±0.38^b^ | 31.47±0.29^c^ | 30.27±0.27^a^ | 29.17±0.33^c^ | 27.07±0.12^d^ | 26.1±0.06^d^ | 24±0.58^c^ | 23.6±0.55^c^ | 22±0.58^d^ |  |
